# Two-Dimensional Materials, the Ultimate Solution for Future Electronics and Very-Large-Scale Integrated Circuits

**DOI:** 10.1007/s40820-025-01769-2

**Published:** 2025-05-13

**Authors:** Laixiang Qin, Li Wang

**Affiliations:** 1 Ningbo Institute of Digital Twin, Eastern Institute of Technology, Ningbo City, 315100 Zhejiang People’s Republic of China; 2 Eastern Institute of Technology, Ningbo City, 315100 Zhejiang People’s Republic of China

**Keywords:** 2D materials, Short channel effects, Integrated circuits, Degraded carrier mobility, Moore’s law

## Abstract

The relentless down-scaling of electronics grands the modern integrated circuits (ICs) with the high speed, low power dissipation and low cost, fulfilling diverse demands of modern life. Whereas, with the semiconductor industry entering into sub-10 nm technology nodes, degrading device performance and increasing power consumption give rise to insurmountable roadblocks confronted by modern ICs that need to be conquered to sustain the Moore law’s life. Bulk semiconductors like prevalent Si are plagued by seriously degraded carrier mobility as thickness thinning down to sub-5 nm, which is imperative to maintain sufficient gate electrostatic controllability to combat the increasingly degraded short channel effects. Nowadays, the emergence of two-dimensional (2D) materials opens up new gateway to eschew the hurdles laid in front of the scaling trend of modern IC, mainly ascribed to their ultimately atomic thickness, capability to maintain carrier mobility with thickness thinning down, dangling-bonds free surface, wide bandgaps tunability and feasibility to constitute diverse heterostructures. Blossoming breakthroughs in discrete electronic device, such as contact engineering, dielectric integration and vigorous channel-length scaling, or large circuits arrays, as boosted yields, improved variations and full-functioned processor fabrication, based on 2D materials have been achieved nowadays, facilitating 2D materials to step under the spotlight of IC industry to be treated as the most potential future successor or complementary counterpart of incumbent Si to further sustain the down-scaling of modern IC.

## Highlights

- With the incessant down-scaling of electronics, traditional semiconductors like Si are encountered with insurmountable hurdles to maintain performance increase without bringing about additional issues of power consumption escalating, in this context, two-dimensional (2D) materials emerge as superior candidates to supersede or complement Si attributed to their marvelous electronic properties to further sustain the Moore’s law life.
- 2D materials-based electronics in More Moore and More than Moore’ regimes have attained promising achievements and showcased monumental potentials applications in low power consumption integrated circuits
- 2D materials-based integrated circuits have gone through a promising development, evolving from small-scale integrated circuits (ICs) to full-functioned processors. Whereas enormous endeavors are waited to be dedicated to realize large-scale ICs attributed to lack of large-scale 2D materials of electronic qualities and immature fabricating techniques.

## Graphical abstract

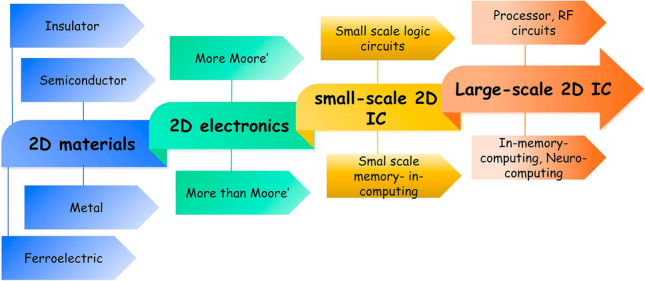

## Introduction

Since Gordern Moore proposed his famous Moore’s law in 1965 [1], integrated circuits doubled every two years, manifesting by physical dimensions of discrete electronic device reducing by 0.7 times, ascribed to which the footprint reduces by ~ 0.5 times. On the contrary, operating speed doubles, power dissipation and cost both decrement by a factor of two [2, 3]. The Moore’s law had been complied with integrated circuits (ICs) happily during the former several decades until semiconductor industry entered into sub-100 nm node, where gate began to give over part of its electrostatic dominance over channel to drain bias attributed to drain’s stealing behavior over channel barrier [4, 5]. In this context, the notorious short channel effects (SCEs) began to come into view and draw themselves enormous attention to be dedicated efforts to [6]. The prominent SCEs that seriously degrade device performance comprise of subthreshold swing (SS), drain-induced barrier lower (DIBL), punch-through, Subthreshold-voltage (*V*_*t*_) rolling off in accordance with channel length scaling and leakage current increasing [7–10]. SS describes the switching capability of the device and the smaller the SS, the stronger the gate electrostatic control over channel is, and DIBL is on behalf of the competing behavior of drain bias over the controllability of channel with regards to gate bias. Punch-through denotes the phenomenon of the depletion region induced by source and drain doping jointing with each other under the channel region, contributing to leakage current increment, while *V*_*t*_ rolling off contributes to leakage current escalating and stability issue in circuits, where leakage current increasing finally results in static power consumption surging. The relentless downscaling of electronics poses imperative requests for novel device structures or ingenious materials which can reach a tradeoff between high discrete device performance and low power dissipation to sustain the Moore’s law life further, this regime of semiconductor technology is termed as More Moore. For another, modern life brings forwards the requirements for 5G or 6G communication, cloud computing, artificial intelligent and neuromorphic computing, which all comes following the blossoming of More than Moore’s regime [11]. More than Moore describes the integrated circuits boost their performance by incorporating of diverse kinds of electronics or optimizing the algorithm to achieve various and enhanced functions [12, 13].

2D materials have spawned dramatic interests ever since graphene was exfoliated in 2004 [14], and are treated as most potential candidates to supersede or complement bulk Si in the foreseeable future in semiconductor industry, which mainly originate from their remarkable electronic properties [15–17]. 2D materials possesses high carrier mobility maintainability regardless of thickness thinned down to sub-1 nm attributing to dangling bonds free and atomic smooth surface, which has long been plaguing bulk semiconductors whose carrier mobility seriously degrades proportionally to the six power of thickness (~ *t*^6^) when thickness decreases to sub-5 nm regime [18]. Nevertheless, it is imperative for channel thickness to thin down proportionally in accordance with channel length to maintain sufficient gate controllability over channel to suppress SCEs according to Dennard’s law [19], thus it seems impossible for bulk semiconductors exclusively to sustain downscaling to keep Moore’s law alive in the coming future. New materials that act as successors or complementary counterparts are indispensable to further push the semiconductor technology nodes forward. 2D materials with atomic thickness, tunable bandgap, large effective mass that are capable of suppressing leakage current and compatibility with both front end of line (FEOL) and back end of line (BEOL) of Si production line come just at the right time, like destined in the fate [20, 21].

2D materials belong to a big family which possess a wide range of electronic properties, covering from insulators (hexagonal boron nitride (h-BN), Bi_2_SeO_5_, SrTiO_3_) [22–24], semiconductors (transition metal dichalcogenides (TMDCs) family) [25, 26], metal (graphene) [27] to ferroelectric materials (In_2_Se_3_, CuInP_2_S_6_) [28], fulfilling the various requirements of diverse electronics or photonics thanks to their potentials for fabricating heterostructures with ultra-sharp van der Waals interfaces, which are prerequisites factors for high performance electronics fabrication [29, 30]. Recently, in several reports, the performance of discrete device based on 2D materials obtained has surpassed that of incumbent Si transistor that has dominated the semiconductor industry long since. Whereas, these devices that show the most cutting-edge performances are generously fabricated by 2D materials exfoliated from the bulk materials, which are of high quality but are restricted in lateral sizes, hindering their application potentials in constituting large scale 2D circuits to be commercialized in semiconductor industry [31, 32]. For another, innovative device structures based on 2D materials that are capable of reducing power dissipation via overthrowing the “Boltzman Tyranny” (The phenomenon that the SS of traditional FET cannot be smaller than 60 mV dec^−1^ stemming from carrier injection transport mechanism) to make it possible to attain sufficient *I*_on_ with lower supply voltage are yet to be optimized [33, 34]. These post Moore’s 2D materials-based ingenious device structures encompass 2D materials-based negative capacitance field effect transistor (NCFET), tunnel field effect transistor (TFET), dirac source FET (DSFET), spin transport FET (S-FET) and impact-ionization (avalenche breakdown) FET (I-IFET), which are capable of obtaining ultra-steep switching behaviors and reducing SS below 60 mV dec^−1^, resulting in diminished supplied voltage required [35–39]. Apart from adopting new physical mechanisms to reduce *V*_*dd*_ for the purpose of lowing down the power dissipation, making full use of the vertical direction to fabricate 3D or vertical device structures is another effective gateway to concurrently achieve sufficient gate controllability to combat with SCEs and reduce leakage current to decrement power consumption. Fin field effect transistor (FinFET) based on Si has proved itself to be deserved the honor and successfully sustain the technology node to 3 nm, though at which SCEs degradation and leakage current increment come back at the revenge [40]. In this context, nanosheet or nanowire Gate all Around FET (NS/NW GAAFET) structures that are treated to possess the ultimate gate controllability arising from the channel being surrounded by gates have come to the rescue, which are projected to further revive the Moore’s law and sustain the technology to sub-1 nm node [41]. Sparked by the ingenious 3D device structures and the SCEs immunity capabilities of 2D materials, 2D materials-based FinFET or NS GAAFET structures have been put forward and fabricated, from which promising performances have been achieved, showcasing the substantial compatibility of 2D materials with modern semiconductor production line [42–44]. More than Moore’s poses the requirements of multi-functions achieved in one single chip, which can be well fulfilled by 2D materials ascribed to the feasibility of 3D integration of 2D materials-based electronics, both with Si or pure 2D circuits. 2D materials-based sensors, memristor, memtransistor, flexible transistors and radio frequency (RF) electronics can be fused into the computing circuits fabricated with Si or 2D materials to facilitate the boosting of multi-functions to fulfill the diverse demands of modern life thanks to the compatibility of 2D materials with BEOL process of modern semiconductor industry and capability of 3D monolithic integration of 2D electronics, offering new opportunities to cram more functional circuits into one chip [45, 46]. Modern life and ICs pose urgent requirements for faster speed, lower cost, and less power consumption day by day, which lay formidable roadblocks for traditional von Neumann structure where larger latency and power consumption have been wasted in shuttling data back and forth from memory region to computing region, that is the notorious “Memory Wall”, which can be ultimately addressed with Non von Neumann structure where computing is conducted in memory regions. 2D materials-based in-memory computing circuits bode well for reducing power consumption, cost and enhancing operating speed with regards to those based on traditional bulk semiconductors [46].

With numerous efforts have been dedicated to the semiconductor technology based on 2D materials, simple logic circuits have sprung up nowadays which showcase superior merits over traditional bulk Si based circuits in footprint reduction, multi-functions obtainment with reduced electronics counts and power consumption. Simple logic circuits based on 2D materials like inverters, NAND, NOR, OR, ring oscillator (RO), static random access memory (SRAM), or amplifiers all have been achieved up to now, which manifested the merits of proper logic functions, power consumption reduction, multi-outputs originating from the ready tunability of electronics states of 2D materials and what is more, the feasibility to reduce electronic device counts by virtue of multi-input ports of 2D materials with double channels [47–51]. Intriguingly, integrated circuits based on 2D materials that encompass divergent functions in one chip have come into view recently thanks to the inexhaustible endeavors dedicated by researchers all over the world, shedding light on the bright future of 2D materials being fused into semiconductor industry. 3D integration of 2D materials-based electronics and simple circuits purely or with mature Si ICs also cast new light on the further miniaturization for More Moore’ or diverse electronic functions incorporation for More than Moore’s in IC technology [52, 53]. Albeit promising as the achievements seems, there are enormous challenges and restrictions for 2D materials-based circuits to be commercially produced some day in the future. Hitherto, most of the 2D materials-based circuits reported are single logic gates, or more complicated, several logic gates being repeated in large arrays, the research works about completed and sophisticated microprocessors or full functional integrated circuits are rare at the moment [54, 55]. The functions of the limited works about the completed circuits are far underperforming than those of Si-based ICs, let alone the low yield, disillusionary operation retention time and poor stability of the circuits. Of course, all these obstacles and challenges are ascribed to 2D materials-based circuits being in the nascent stage, which are common for all the new semiconductor materials who are about to step onto the history stage of the semiconductor industry.

This review briefly outlines the inevitable trend of 2D materials being treated as the most potential candidates in future VLSI from below several aspects. Firstly, a comprehensive introduction part give the readers the impression that the further sustainability of modern IC calls for new kinds of novel materials to succeed or complement bulk semiconductors, and fortunately, 2D materials come to the rescue just at the right time; the second part introduces the 2D materials in detail, covering the main 2D materials families according to their electronic or photonic properties; the third part summarizes several main electronic devices based on 2D materials, comprising of their physical mechanisms and current cutting-edge breakthroughs both from more Moore and More than Moore regimes; the fourth part give a brief introduction of modern IC based on 2D materials achieved by researchers both from academia and industry, covering from discrete basic cornerstone inverters, small-scale logic circuits, to large-scale full functional circuits; the final part summarized the challenges and opportunities encountered by 2D materials to be incorporated into the modern IC as a successor or complementary counterpart to bulk semiconductors, the challenges include materials synthesis, discrete device performance boosting, yield improvement and variations decrement of large scale device arrays in IC based on 2D materials. Of course opportunities and hopes exist, blossoming ICs both of more Moore and more than Moore’s based on 2D materials which are characterized by ultra-high operating speed, ultra-low power consumption, substantially decreased cost and diverse functions that fulfill the envisions of future life are awaiting somewhere in the future not that far away as soon as the challenges mentioned above are conquered.

## Two-Dimensional Materials

2D materials have spawn dramatic interests recently attributed to their marvelous electronic properties and a wide range of materials properties covering from insulator, semiconductor to metal which are readily to fabricate various kinds of van der Waals interfaces for different electronic or photonic purposes [56, 57]. Hitherto, there are several kinds of 2D materials that have been experimentally demonstrated, which are mainly classified into below several family groups who possess their own unique properties: (1) transition metal dichalcogenides (TMDCs), (2) black phosphorus (BP), (3) hexagonal boron nitride (h-BN), (4) mono-element compounds (Xenes), (5) III-IV Transition Metal Chalcogenide and the like. New kinds of 2D materials keeps on emerging thanks to the relentless efforts devoted by researchers both from simulation and experimental regimes, offering a plethora of platforms for various kinds of electronics or photonics [17, 58, 59].

### Transition Metal Dichalcogenides

TMDCs are a group of materials which are intensively studied and demonstrate paramount promises in the regimes of electronics, photonics, sensors and artificial intelligence which have attracted immense interests by virtue of their atomically thin thickness, varying bandgaps between 1 and 2 eV, which can fulfill the requirements of various applications, dangling-bonds free surfaces and relatively larger effective carrier masses that can contribute to leakage current suppression [60]. MoS_2_ is the most representative 2D TMDC material whose monolayer form was firstly exfoliated in 1966 by Frindt with scotch tape [61], and was chemically exfoliated 20 years later [62]. Monolayer MoS_2_ has a direct bandgap of 1.8 eV, and gradually reduces to an indirect bandgap of 0.4 eV as when it is in bulk form. Thanks to its environmental stability, 2D MoS_2_ has been treated as the most potential supersedure or complement to 3D Si in the post Moore’s era and widely investigated by researchers both from academia and industry. In the typical crystal structure of monolayer 2D MoS_2_, a molybdenum atom lies between two sulfide atoms, jointed together via covalent bonds, constituting a monolayer of 6.5 A. Whereas, different MoS_2_ layers are jointed by van der Waals force, which bodes well for its monolayer 2D form to be exfoliated mechanically or chemically [63]. The burgeoning achievements and bright foreground brought about by 2D MoS_2_ ignite immense interests in delving into 2D TMDCs materials manifested by a formula of MX_2_, where M stands for transition metal (for example, Mo, W, Re or Ta) who locates between two X atoms which is on behalf of the chalcogen family element (taking S, Se, Te, and O as examples), jointed by covalent bonds. Just like their counterpart MoS_2_, other 2D TMDCs materials are characterized of direct bandgaps in monolayer, which gradually decrease and change to indirect bandgaps with increasing thickness [64–66]. Attributed to their tunable bandgaps in a reasonable range and relatively large effective carrier mass, 2D TMDCs materials are generally hailed as suitable channel materials in electronics or photonics. In 2011, monolayer MoS_2_ was firstly used in a transistor as a channel materials with carbon nanotube as gate, high on/off ratio of exceeding 10^8^, high mobility of 200 cm^2^ V^−1^ s^−1^ and near ideal SS of 74 mV dec^−1^ had been obtained, perfectly approaching the ideal 60 mV dec^−1^ limit for conventional transistor at room temperature, boasting their feasibility in suppressing short channel effects deriving from 2D materials’ ultimate thickness [67]. From then on, tremendous efforts have been dedicated to this research regimes and various stable 2D TMDCs materials have been experimentally substantiated which cover wide range of electronic properties from semiconductor, semimetal, insulator to superconductors attributed to their crystal structures or different constituting M or X elements [65]. The most prevalent 2D TMDC materials that are used as channel materials can be categorized as n type semiconductors as MoS_2_, MoSe_2_, or WS_2_, p type semiconductors like MoTe_2_ and WSe_2_ who can demonstrates ambipolar transport behavior stemming from its specific band structure [68–70]. Crystal structures have the last words for the electronic properties of specific 2D TMDC materials, which commonly comprise of 2H, 1 T, 1 T’ phase structures depending on their fabrication conditions. 2H phase TMDC materials own the crystal structure of trigonal prismatic, demonstrating semiconductor behavior, while 1 T or 1 T’ phases manifest metallic behavior, offering an additional knob for phase engineering to reduce contact in 2D TMDC materials based electronics [71, 72].

The merits of 2D TMDC semiconductors to be used as channel materials in post Moore’ regime lies in their carrier mobility maintenance as thickness reduces to sub-1 nm, which has been plaguing 3D semiconductors whose carrier mobility degrades substantially following the six power law as thickness reduce down to below 5 nm [73]. The reported carrier mobility of 2D TMDC materials lies around 200 cm^2^ V^−1^ s^−1^, which is consistent with that of bulk Si, holding colossal promises to be applied in Si based semiconductor industry. Nevertheless, the carrier mobility of 2D TMDC materials can be seriously affected by interface materials and quality, thus carrier mobility of monolayer 2D TMDC materials is often significantly degraded than their multilayer or bulk forms attributed to the fact that monolayer 2D materials are more susceptible to interface states due to their ultimate thickness [74]. Encapsulation by h-BN or inserting other buffer layer or introducing strain in 2D TMDC materials is capable to help to reduce the impact of interface states and increase their carrier mobility. Newly found 2D TMDC materials of ReS_2_, NbSe_2_ or TaS_2_ show superior semiconductor or superconductor behaviors, offering additional platforms for 2D TMDC materials to be used in fundamental research or new kinds of functional devices.

### Black Phosphorus

Black phosphorus (BP) is a typical p type 2D semiconductor with a direct bandgap varying from 0.3 to 2.0 eV as its thickness reduces from bulk to monolayer. In BP crystals, each phosphorus atom is covalently bonded via *sp*^3^ hybridization to three neighbor phosphorus atoms, forming an orthorhombic crystal structure, leading to its semiconductor behavior while different BP layers are jointed by van der Waals force [75]. The crystal structures in x and y directions are also asymmetric, leading to different carrier mobility along armchair and zigzag directions. Albeit it is unstable in atmosphere, its high carrier mobility (over 1000 cm^2^ V^−1^ s^−1^ in monolayer at room temperature and more than 70,000 cm^2^ V^−1^ s^−1^ in bulk form) still wins itself enormous trials to be used in electronics or photonics [76]. 2D BP materials are potential candidates in high performance logic electronics ascribed to their high carrier mobility and relatively modest bandgaps. A plethora of reported transistors with recorded high on state current and transconductance are made from BP materials [77–79]. Besides, thanks to their readily tunable direct bandgaps and high carrier mobility, 2D BP materials show tremendous potentials in photonics or detectors [80–82]. The prominent roadblock confronted by 2D BP materials to be applied in high performance electronics or photonics is their environmental instability, which endows enormous challenges in fabricating 2D BP based devices stemming from their rapid degradation in environment. Encapsulation via h-BN, oxide materials, or fabricating 2D BP-based devices in vacuum atmosphere or glove box can alleviate the hurdle to some extent, whereas, radical avenues should be opened up to surmount the challenges ultimately [83, 84]. For another, the readily tunable direct bandgap of BP also provides an additional knob in forming homojunctions that can contribute to sharp and ultra-clean junctions in tunnel field effect transistor, leading to boosted *I*_on_ without compromising steep switch behavior [85].

### III-IV Transition Metal Chalcogenide

III-V transition metal chalcogenides which are manifested by MX or MX_2_ (M possesses the elements of gallium (Ga), indium (In), tin (Sn), thallium (Tl), lead (Pb), and bismuth (Bi)) and X comprises of S, Se, Te, and O) are newly emerging transition metal chalcogenide 2D materials, among which Bi-based 2D chalcogenide materials often behave as topological insulator, while Pb- or Tl-based chalcogenide materials demonstrate as three components, which are rarely considered to be used in semiconductor industry [86]. Ga-based chalcogenides materials comprising of GaS, GaSe, GaTe demonstrate relatively different properties in contrast to 2D TMDC materials, which are manifested by larger bandgaps, with bulk materials possessing bandgaps ranging from 1.7 to 2.5 eV and monolayer demonstrating bandgaps varying from 3.3 to 3.4 eV [87]. The relatively large bandgaps of Ga-based chalcogenides materials offer potential opportunities in being used in optoelectronics working in UV to visible spectrum range. For another, 2D Ga-based chalcogenides materials possess unusual band alignments, resulting in larger effective carrier mass, which contributes to leakage current suppression in ultra-scaled field effect transistors. Unfortunately, attributing to their larger bandgaps and effective carrier mass, carrier mobility is constrained, which lays aside substantial challenges for 2D Ga-based chalcogenides materials to be applied in high speed electronics [88]. Recently, 2D InSe material triggers enormous attentions arising from its high carrier mobility and light effective mass (~ 0.14 m_0_). A record high electron mobility exceeding 3700 cm^2^ V^−1^ s^−1^ had been obtained in InSe-based FET [89], which gained to 10^4^ cm^2^ V^−1^ s^−1^ in Hall structure via encapsulated by BN and contacted by graphene at liquid-helium temperature [90]. A ballistic 2D transistor with 10 nm channel length made from exfoliated InSe had been reported, the transistor exhibited a record high *I*_on_ of more than 1 mA μm^−1^, an ohmic contact of 62 Ω μm^−1^, SS of 76 mV dec^−1^ and *I*_on_/*I*_off_ ratio over 10^8^, a DIBL of 22 mV V^−1^, indicating perfectly suppressed SCEs [31]. The high mobility and relatively modest bandgap of 1.2 eV make InSe to bode well for high performance logic electronics.

Aside from group III element, group IV element as Sn-based chalcogenide 2D materials have ignited immense attentions thanks to their marvelous properties. SnSe is treated as conspicuous candidate for thermoelectrics attributed to its ultra-low lattice thermal conductivity and high thermalelectric figure of merit [91]. By virtue of its low bandgap of 0.86 eV and small effective carrier mass (0.14 m_0_ and 0.08 m_0_ for zigzag and armchair direction), 2D SnSe materials show promise in high speed electronics. A best reported 2D SnSe-based transistor demonstrated a high electron mobility of more than 250 cm^2^ V^−1^ s^−1^, and a on state current of 0.3 mA μm^−1^ with a superior on/off ratio of more than 10^7^, providing an additional platform for high speed electronics except for 2D BP, while getting rid of environmental instability issue at the same time since SnSe-based transistor was reported to be capable of maintaining its performance for more than three months, which had been plaguing 2D BP long since [92].

### h-BN and Other 2D Insulator Materials

2D materials-based electronics have been confronted with the challenges of high κ material integration, which has been alleviated more or less thanks to the emerging and incorporation of 2D dielectrics. Hexagonal boron nitride (h-BN) is widely studied and mostly representative 2D dielectric material with a relatively large bandgap of 6 eV and dielectric constant of ~ 5. The crystal structure of h-BN is characterized by hexagonally linked *sp*^2^ hybridized B–N bonds, h-BN is capable of constituting ultra-clean van der Waals interface with 2D semiconductor channel, which is beneficial for leakage current suppression and switching speed improvement, whereas, the low dielectric constant of h-BN hampers its capability to be reduced to sub-1 nm equivalent oxide thickness (EOT), preventing it from being used in ultra-scaled electronic devices [93]. An alternative pathway is to adopt h-BN in combined with other high κ dielectric materials as HfO_2_, Al_2_O_3_ or ZrO_2_, where h-BN behaves as an interlayer which helps to reduce the interface states between 3D high κ dielectrics and 2D semiconductor channels [94]. Generally, the combined h-BN/high κ oxide dielectrics attest to outperform pure high κ oxide layer on 2D semiconductor channel. For another, ascribed to its robust environmental stability and marvelous flexibility, h-BN is prevalently used as encapsulation materials for 2D materials-based electronics, especially those who are sensitive to oxygen or moisture in atmosphere, such as BP, InSe, or MoTe_2_ [95]. 2D materials as graphene or MoS_2_ encapsulated with h-BN proved to be feasible to enhance their carrier mobility substantially, the reason lies in the merits of mechanical flexibility and scalability of h-BN contributes to the scattering suppression from substrates [96]. h-BN can also serve as interlayer between 2D source/drain materials and 3D metal layer, contributing to unpinning Fermi-Level and impeding metal induced gap states (MIGS), suppressing trap states and avoiding inter-diffusion between contact metal and 2D materials, promising achievements have been demonstrated which consolidate the efficacy of h-BN in helping to reduce contact resistance in 2D materials-based electronics [97].

Whist 2D h-BN possesses the feasibility of forming ultra-clean interfaces between dielectric materials and 2D channels, its low permittivity limits its application in ultra-scaled electronic devices. Thus pursuing for 2D dielectric materials with high dielectric constants is imperative for 2D material based electronics to achieve high performance. Fortunately, hard work pays off ultimately. Recently, 2D van der Waals or quasi 2D van der Waals dielectrics keeps on coming into our view in the regime of 2D materials-based electronics, among which Bi_2_SeO_5_ and SrTiO_3_ are the most promising candidates [98, 99]. Bi_2_SeO_5_ is generally obtained by layer by layer oxidation of Bi_2_O_2_Se, which is a newly fabricated 2D material with high mobility. Deriving from the feasibility of gradually transition of 2D Bi_2_O_2_Se semiconductor material to 2D Bi_2_SeO_5_ insulator who possesses high dielectric constant of 21, transistors based on 2D Bi_2_O_2_Se as semiconductor channel and its corresponding high κ native oxide 2D Bi_2_SeO_5_ as gate dielectrics are promising in achieving high performance by virtue of the ultra-sharp and clean interface between channel and dielectric layer, impeding the interface trap states that can lead to high leakage current and degraded switching behavior. A field effect transistor based on such a material system demonstrated mobility higher than 300 cm^2^ V^−1^ s^−1^, on/off ratio over 10^5^ and low SS of ~ 75 mV dec^−1^ at room temperature attributed to the ultra-clean interface between 2D channel and gate dielectric layer, based on which inverters circuits showcased voltage gain as high as 150 [100].

### Graphene, Germanene and Silicene

Graphene was firstly mechanically exfoliated with scotch tape in 2004 by Novoselov and Geim [14]. The emerging of graphene has opened up a new materials platform for both fundamental research and novel electronic or photonic devices, since when diverse 2D materials keep on blossoming and burgeoning with marvelous achievements having been attained. Graphene is a metallic 2D material with carbon elements arranged in a hexagonal structure, which contributes to its novel dirac cone band alignment and environmental stability, making it a super candidate for electrode in various kinds of devices [101]. The carrier mobility in graphene is high, over 10^4^ cm^2^ V^−1^ s^−1^ at environment and can reach 10^5^ when suspended [102]. Albeit the carrier mobility in graphene is extremely high, the absence of bandgap prevents graphene from being used in logic devices, which raises a claim for at least 500 mV bandgap to obtain an on/off ratio at any rate over 10^4^–10^7^ to circumvent code ambiguity [103]. Narrowing nanoribbon width provides additional knob to open a bandgap in graphene and transistors based on nanoribbon graphenes have been reported more or less, whereas, narrowing the width of graphene nanoribbon has been faced with massive challenges which encompass both experimental technique limitations and electronic properties degradation [104]. Thus graphene steers its wheel and finds ample scope for its skills that is radio frequency (RF) devices that have no requirements for bandgaps in achieving high cutoff frequency. Graphene-based RF devices demonstrates a high cutoff frequency of hundreds of GHz, only that the oscillation frequency which is key in circuit application remains low, which puts constraints on the application of graphene [105]. Besides, attributed to its superior conductivity and flexibility, graphene has been widely used as electrodes materials in both 2D materials-based electronics and photonics, where the ultra-clean van der Waals interfaces benefit for the alleviation of Fermi level pinning issues in contacts regions [101].

Silicene and germanene are allotropes of the modern semiconductor backbones of silicon and germanium, which also feature hexagonal honeycomb crystal structures alike that of graphene. Different from the fact that A and B sublattice locates in the same plane, the sublattices in silicene and germanene have a perpendicular angle to the plane, which contributes to a minor bandgap of 1.55 and 23.9 meV in silicene and germanene, shedding lights on their usages in digital logics [106]. For another, the asymmetric crystal structures also offer more opportunities for silicene and germanene to further enlarge their bandgaps via strain, applying vertical electric field or adopting other avenues [107]. The high theoretically predicated mobility of 10^4^–10^5^ cm^2^ V^−1^ s^−1^, feasibility in further tuning bandgaps and being allotropes of backbone channel materials of silicon and germanium make the two 2D silicene and germanene attractive to researchers in spite of their instability and huge challenges in materials synthesis. Simply put, 2D silicene and germanene are promising candidates for high performance logic electronics thanks to their high carrier mobility despite of the pronounced hurdles lay in front of the massive production of relevant electronics made from them.

## Electronics Based on Two-Dimensional Materials

With the incessant downscaling of electronics, traditional 3D semiconductors have encountered paramount physical limitations in further scaling down channel thickness to fulfill the requirements of enough gate electrostatic controllability to hinder the increasingly degraded SCEs and worsen leakage currents. The emerging of various 2D materials opens up new avenues for further miniaturization of electronic dimensions deriving from the feasibility of 2D materials in maintaining high carrier mobility with channel thickness thinning down [32]. With numerous endeavors being devoted to the investigation of 2D materials, a myriad of achievements have been achieved in the regime of 2D materials-based electronics, comprising of electronics both from More Moore’s and More than More’s regimes [108], which will be comprehensively discussed in the below sections to give readers a holistic impressions.

### Electronics for More Moore’s

**Fig. 1:**
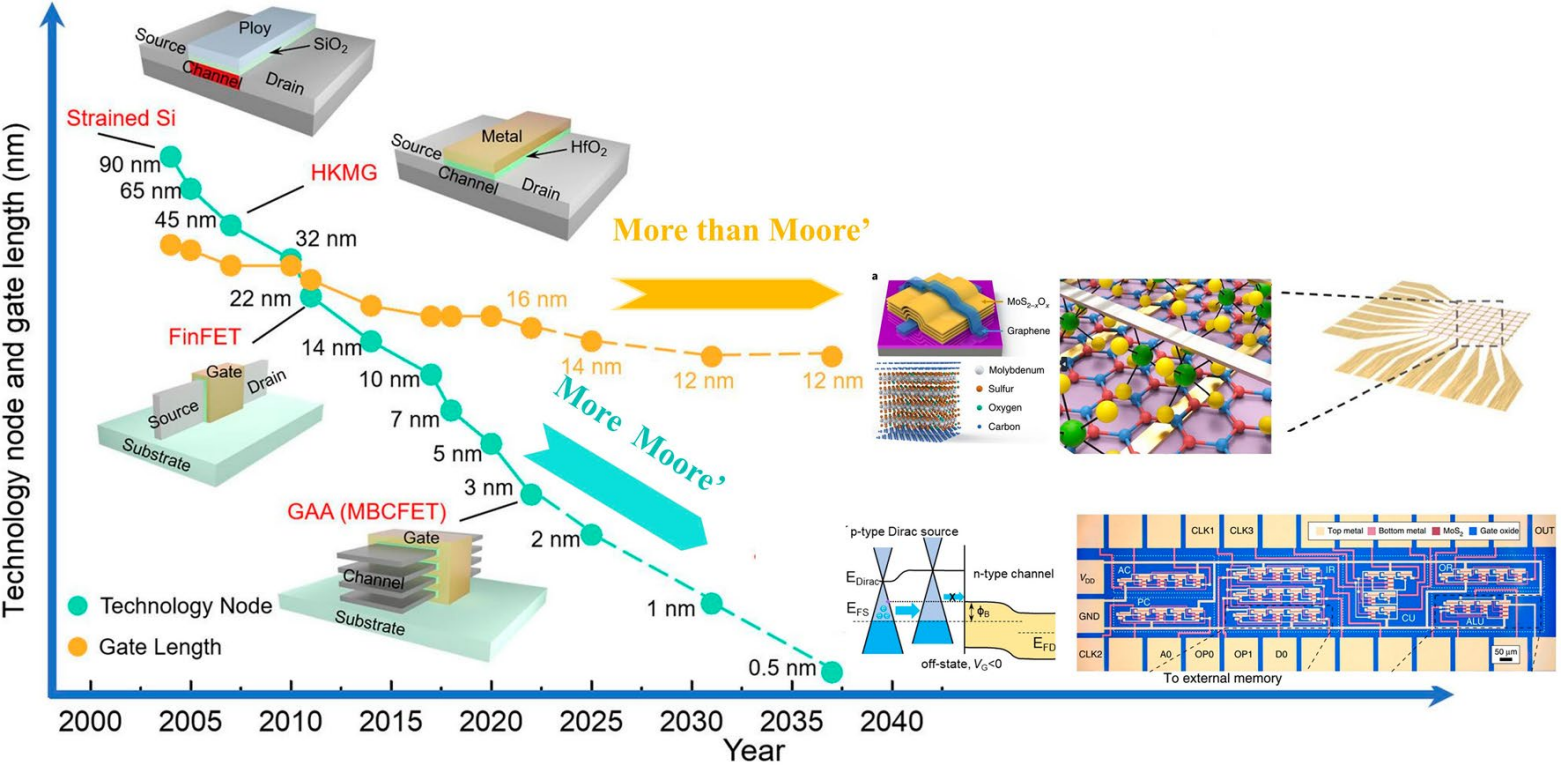
Evolving trend of Integrated Circuits with time, one is more Moore’s and the other is more than Moore’s, reprinted with permission from Ref. [12, 150, 194, and 256]

**Fig. 2:**
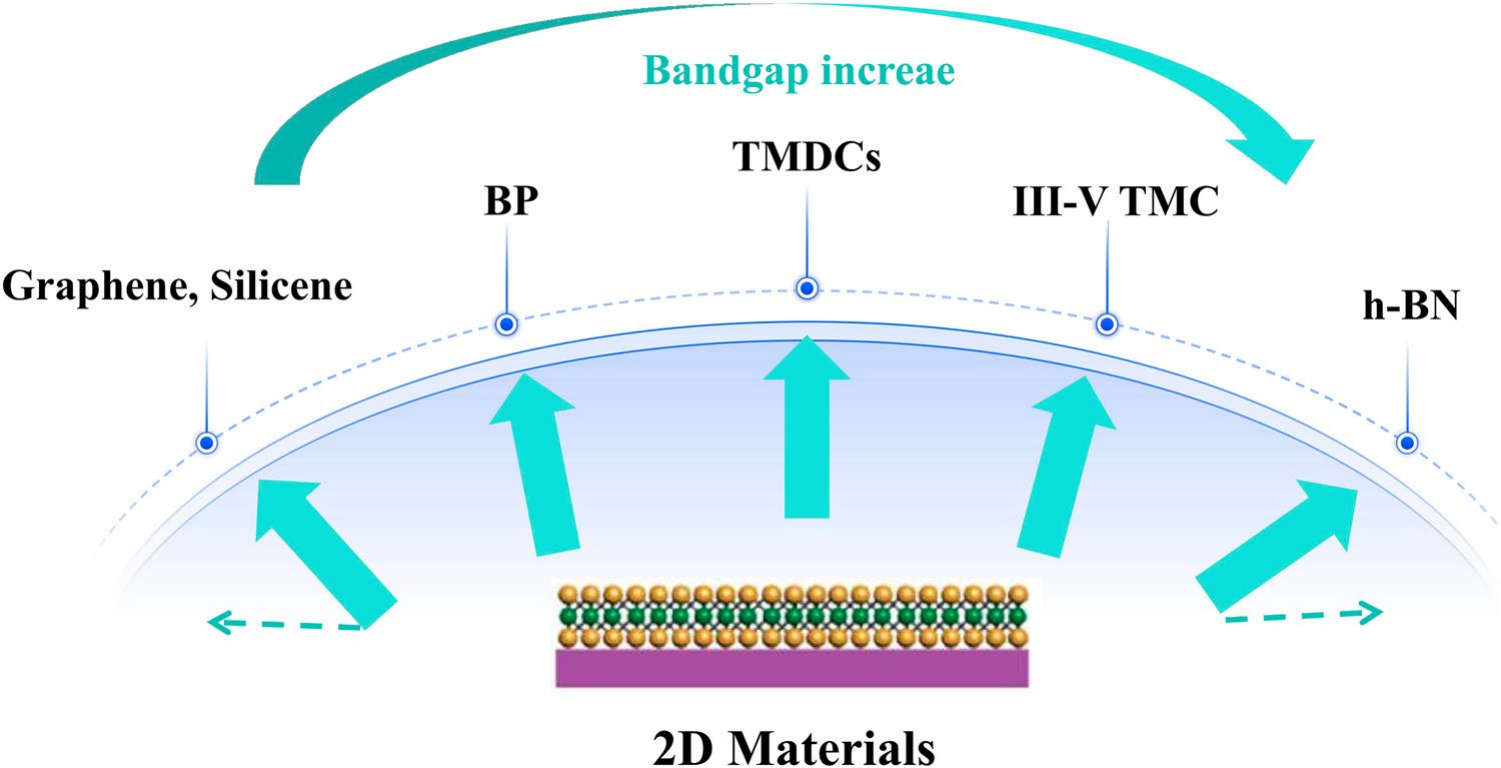
Classifications of 2D materials, mainly comprising of metallic graphene, germanene, and silicene; BP; TMDCs; III-V TMC and insulator h-BN

More Moore’s regime denotes the era when traditional electronics encounters plateau in its scaling trend, which needs to be surmounted via adopting new materials or steering to novel device structures. The electronics in More Moore’s regime are characterized by enhanced device performance in the context of marginal power consumption, which encompass logic devices and memory devices as well. In logic devices, in order to improve computing speed with dimension downscaling, electronics with new working mechanism, new channel materials or new device structures are desperate for transformative breakthroughs to combat the increasingly degraded SCEs and leakage currents. 2D materials-based electronics of new mechanism comprise of steep slope transistors as 2D materials-based tunnel field effect transistor (TFET), negative capacitance field effect transistor (NCFET), dirac source field effect transistor (DSFET), spintronic field effect transistor (S-FET), ionic impact field effect transistor (I-IFET) as mentioned above. New channel materials mainly focus on 2D materials and carbon nanotubes which stand out among many candidates attributed to their superior device performance and lower power consumption; For another, 2D materials combined with the incumbent prevalently SCEs suppression device structures as FinFET and GAAFET have spawned enormous interests as well. In memory device, except for reducing memory device dimensions to reduce the footprint of memory region to contribute to chip minimization, power consumption of memory devices is poised to be diminished to contribute to the overall power consumption decrement in modern IC chip. 2D material based memory devices possess the feasibility to reduce the supply voltage needed, resulting in diminished power consumption. For another, attributed to the atomic flat surface and transferability of the 2D materials, it is feasible to fabricate double or even multi-gates 2D materials-based electronics, benefiting for providing more inputs or outputs ports, contributing to electronic devices counts reduction in 2D materials-based memory modules, resulting in area-efficiency and power consumption inhibited ICs (Figs. 1 and 2).

#### Computing Devices

The ceaseless miniaturization of electronics presents relentless requirements for power consumption decrement in the context that device performance keeps on elevating, posing enormous challenges to further scale down supply voltage in accordance with electronic dimensions, which are leveling off for a long period of time ascribed to the notorious “Boltzman Tyranny” in traditional transistors [109]. “Boltzman Tyranny” denotes the phenomenon that the SS of transistors is incapable of being reduced down below 60 mV dec^−1^ at room temperature stemming from the carrier injection mechanism, which prevents V_DD_ in traditional transistors from being further scaled down, hindering the reduction of power dissipation. Fortunately, the new kinds of electronics FET devices incorporating of TFET, NCFET, DSFET, and I-IFET can fulfill the demands of reducing V_DD_ without trading off device performance via ultra-steep switching phenomena, that is, SS below 60 mV dec^−1^ [110]. What is more, the combination of steep slope FET with novel 2D materials has opened up new avenue for overcoming the fundamental limits of traditional 3D semiconductors based steep slope FETs and expediting commercialization of 2D materials-based electronics [33].

##### 2D TFET

TFET has undergone a long history, whereas, during which TFETs has been plagued by low on state current ascribed to carrier transporting by band-to-band tunneling (BTBT) mechanism. For another, traditional 3D semiconductors based TFET has been encountered with the hurdles of inferior interfaces laden with trap states which results in leakage current increment, intunable band diagrams, wide tunneling barriers for charge carriers, all of which contribute to *I*_on_ inferiority in TFET, which is several orders of magnitude smaller than that of traditional MOSFET, posing insurmountable obstacles for 3D semiconductors-based TFETs to be commercialized [111].

The *I*_on_ in TFET is dominantly determined by the tunneling probability of charge carriers, which can be described as *T*_WKB_. The BTBT in TFET can be comprehensively investigated via a Wentzel–Kramer–Brillouin (WKB) approach which defines the tunneling barrier as a triangular shape [112]. According to the WKB approximation, the *T*_WKB_, which defines the likelihood of a charge carrier to tunnel from source to channel via transmitting through the triangular barrier, can be described as below:

1 $${\text{T}}_{{{\text{WKB}}}} \approx \exp (\frac{{ - 4\lambda E_{{\text{g}}}^{3/2} \sqrt {2m_{\text{t}}^{*} } }}{{3q\hbar (E_{{\text{g}}} + \Delta \phi )}})$$

*m*_t_* denotes the tunneling mass; *q* is the electronic charge; *E*_g_ represents the bandgap; ℏ defines the reduced Plank constant; Δ*φ* is on behalf of the energy difference between the source valence band maxima (VBM) and channel conduction band minima (CBM); *λ* refers to the short channel screen length which is determined by the gate electrostatic controllability over channel. According to Eq. (1), a high tunneling probability, *T*_WKB_, which can guarantee a higher *I*_on_, is facilitated by a smaller tunneling mass *m*_t_*, screen length *λ* and larger Δ*φ* [113].

**Fig. 3:**
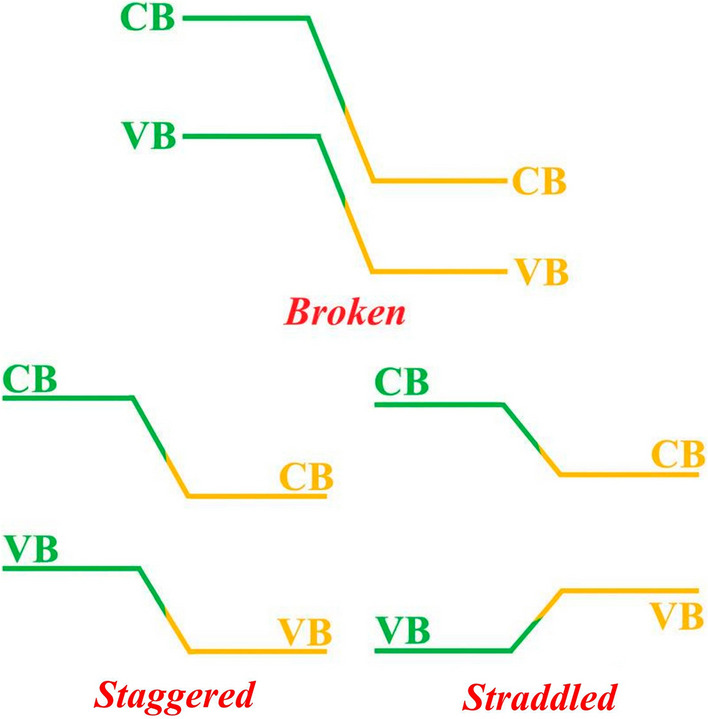
Schematics of straddled (*type I*), staggered (*type II*) and broken (*type III*) band diagrams [33]

Attributed to the inferior interfaces brought about by 3D semiconductor materials, larger *I*_on_ with sub-60 mV dec^−1^ SS in 3D TFET face insurmountable challenges, while fortunately, the emergence of 2D semiconductors has brought in good news for TFET structures, which have been relegated to limbo imputing their infeasibility to improve *I*_on_. 2D materials cover a wide range of material types, from insulator, semiconductor to metals, which comprise of a wide range of bandgaps, holding the promise of constituting various kinds of band diagrams to provide the platform for TFET to choose the most favorable one to boost *I*_on_. Three common band diagrams are generally formed between heterojunctions constituted by two different kinds of semiconductors, which comprising of staggered, broken and straddled band diagrams, among which the staggered and broken band diagrams are favorable for 2D TFETs, as shown in Fig. 3 [33]. The feasibility of constituting various homo- or heterojunctions between different 2D semiconductor materials opens up new avenues for TFET to obtain low SS and high *I*_on_ concurrently without compromising power consumption. A myriad of 2D materials-based heterojunction TFETs have been reported with sub-60 mV dec^−1^ SS and intriguingly higher I_on_ that shows promise for fulfilling the requirements set by International Roadmap for Device and System (IRDS) [114–118]. To overcome the density of states (DOS) inferiority in 2D materials, some 3D semiconductors of Si, Ge or 3D BP have been chosen as source materials to constitute a 3D/2D source/channel heterojunction TFET structures, which benefit for superior I_on_ deriving from highly doped 3D source materials and elevated DOSs [119–121]. A relatively highly doped source with narrow bandgap and intrinsic channel with larger bandgap are preferred in 2D TFET device structure design for higher *I*_on_ and suppressed *I*_off_ concurrently [121]. For another, 2D semiconductors are featured by easily tuned bandgaps via varying thickness, laying the foundation for constituting ultra-clean homojunctions via the same kinds of materials with different thickness [122, 123]. 2D TFET holds the promise of subduing the hurdles confronted by conventional 3D TFET and achieving higher I_on_ and steep switch simultaneously, paving the way for TFET to be commercialized someday in the future.

##### 2D NCFET

NCFET is another potential “green” transistor with steep switch behavior. By incorporating an additional ferroelectric materials layer during the fabrication of gate electrode, internal voltage amplification effect can be introduced into NCFET structure which contributes to steep switch behavior from on state to off state in NCFET with *I*_on_ boosted up synchronously. Different from TFET which depends on adopting new charge carrier transport mechanism to obtain sub-thermionic SS, NCFET demonstrates steep switch phenomenon via making use of the negative capacitance effect of ferroelectric materials inserted in the gate dielectric layer to boost up charge deviation caused by voltage variation, resulting in body factor m to be smaller than 1, leading to sub-60 mV dec^−1^ SS according to Eq. (2), where m is on behalf of the body factor that describes the charge deviation endowed by voltage changing; n is termed as the transport factor that signifies the drain current altering behavior induced by channel surface potential variation; *φ*_S_ is on behalf of surface potential of the channel; *C*_S_ manifests the capacitance of semiconductor transistor below gate oxide, while *C*_gate_ signifies the capacitance of the gate, encompassing the combination of dielectric and ferroelectric capacitance [124];

2 $$\begin{gathered} {\text{SS}} = \frac{{\partial V_{G} }}{{\partial \log_{10} I_{D} }} = \frac{{\partial V_{G} }}{{\partial \phi_{{\text{S}}} }} \cdot \frac{{\partial \phi_{{\text{S}}} }}{{\partial \log_{10} I_{D} }} = m \cdot n \hfill \\ m = \frac{{\partial V_{G} }}{{\partial \phi_{{\text{S}}} }} = (1 + \frac{{C_{S} }}{{C_{{{\text{gate}}}} }}) \hfill \\ n = \frac{{\partial \phi_{{\text{S}}} }}{{\partial \log_{10} I_{D} }} = \frac{{k_{B} T}}{q}\ln (10) = 60{\text{mV/dec}} \hfill \\ \end{gathered}$$

**Fig. 4:**
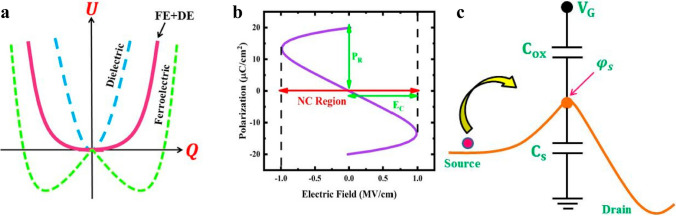
**a** Free energies versus charge carriers of ferroelectric, dielectric, and ferroelectric/dielectric heterostructure; **b** S shaped polarization versus electric field curve of ferroelectric material; **c** Capacitance equivalent circuit of NCFET, the C_OX_ comprises of ferroelectric and dielectric materials [126]

NCFET holds the merits of compatibility with modern semiconductor manufacturing procedure, substantiated by NC-FinFET-based logic circuits demonstrated superior performance than FinFET of the same technology node, which was checked by Wei-Xiang You et al. with the aid of a short-channel NC-FinFET compact model in 2019 [125]. Promisingly as NCFET has showcased in commercial application, hysteresis behavior poses a paramount hurdle in the application of NCFET, aside from a potential instability issue; inability to scaling down ferroelectric materials in proportional to device dimension diminishing and so forth [126]. Fortunately, the hysteresis behavior can be suppressed to a pronounced extent by capacitance matching imposed by tuning capacitance of ferroelectric materials to match well with that of the MOSFET to make sure that the ferroelectric materials work stably in negative capacitance region by confirming that the Gjbbs energy of the composite system comprising of ferroelectric material and dielectric materials possess one lowest energy at zero voltage point, as demonstrated by Fig. 4a. The ferroelectric capacitance, *C*_fe_ that can fulfill the expectation of hindering hysteresis behavior and minimizing SS as much as possible must meet the conditions as below according to Fig. 4c [126], − 1 < *C*_S_/*C*_*OX*_ < 0, where *C*_*OX*_ is the total capacitance of dielectric and ferroelectric materials and C_S_ is the capacitance of transistor and is always positive. To guarantee the NCFET to function without hysteresis and possess steep switch behavior, |*C*_*OX*_| should be larger than *C*_S_, and the more the |*C*_*OX*_| is near to *C*_S_, the smaller the SS is. Once |*C*_*OX*_| is substantially larger than *C*_S_, the internal voltage amplification effect gets almost smeared, resulting in negligible steep switching behavior.

NCFET can be categorized either by ferroelectric materials or via gate stack structures. Ferroelectric materials can be divided into organic, perovskite, HfO_2_-based and 2D ferroelectric materials, among which HfO_2_-based ferroelectric materials demonstrate promising potential to be used commercially attributed to the fact that HfO_2_ is the prevalent high κ oxide materials used in modern semiconductor industry, while 2D ferroelectric materials who emerged recently demonstrate potential in 2D NCFET to impede surface interface states that lead to both high leakage current and smeared steep switch behavior [127, 128]. For another, gate stacks encompass metal-ferroelectric-semiconductor (MFS), metal-ferroelectric-insulator-semiconductor (MFIS) and metal-ferroelectric-metal–insulator-semiconductor (MFMIS) structures, among which MFIS gate stack stands out deriving from both relatively simple manufacturing process and superior device performance [129].

2D NCFET combine the merits of both NCFET and 2D materials to guarantee the restless minimization of electronic devices. The commonly used ferroelectric materials in 2D NCFET encompass organic ferroelectric materials, HfO_2_-based ferroelectric materials and recently emerged 2D ferroelectric materials [130]. The delicate interfaces between 2D semiconductor channels and 3D ferroelectric materials often result in massive trap states that can smear out the ultra-steep switch behavior, leading to prodigious challenges in achieving sub-60 mV dec^−1^ SS in 2D NCFET [131]. A 2D NCFET with organic ferroelectric materials poly(vinylidene difluoride-trifluoroethylene) (P(VDF-TrFE)) combined with 2D MoS_2_ or MoSe_2_ channels obtained an ultra-low SS of 24.4 mV dec^−1^ and sub-60 mV dec^−1^ SS over four orders of magnitudes drain current under a drain bias of 0.1 V [132]. Attributable to the prodigious potential in commercial application of HfO_2_-based ferroelectric materials, 2D NCFETs based on HfO_2_-based ferroelectric materials have garnered monumental attentions. Mengwei Si and coworkers for the first time fabricated a 2D NCFET based on hafnium zirconium oxide (HZO) and 2D MoS_2_ channel, a minimum SS of 5.6 mV dec^−1^ and maximum I_ds_ of 510 uA um^−1^ with extraordinary on/off ratio had been attained [133]. By transferring MoS_2_ channel on top of metal-hafnium zirconium oxide (HZO) ferroelectric-metal structure and varying ferroelectric material thickness, Felicia A. McGuire achieved a minimum SS of 6.07 mV dec^−1^ with an average SS of 8.03 mV dec^−1^ over 4 orders of magnitude *I*_*ds*_, a *V*_th_ shift in accordance with source/drain overlap capacitance and HZO or HfO_2_ thickness variation, demonstrating promising potential in dimensional and *V*_th_ scaling in 2D NCFET [134]. 2D NCFET based on 3D perovskite ferroelectric material also achieved sub-60 mV dec^−1^ SS by marginally tuning 2D NCFET device structure [135]. Albeit 2D NCFETs with HfO_2_-based ferroelectric materials have witnessed promising achievements, the obstacles in attaining ultra-clean interfaces between 3D ferroelectric materials and 2D channels are still desperate for efforts to be devoted to by researchers to solve. In this context, the discovery of 2D ferroelectric materials is just in time, holding the promise of forming ultra-clean interfaces between ferroelectric material and 2D semiconductor channels with trap states enormously hampered [136]. CuInP_2_S_6_ (CIPS) and *α*-In_2_Se_3_ are widely used 2D ferroelectric materials in 2D NCFET [137]. Xiaowei Wang and coworkers demonstrated a 2D CIPS-based van der Waals 2D NCFET with SS below Boltzmann’s limit for 7 decades of I_ds_ and minimum SS of 28 mV dec^−1^, where the expanded scope of I_ds_ for sub-thermionic SS compared with 3D ferroelectric materials based 2D NCFET originated from the ultra-clean interface between 2D CIPS and below 2D semiconductor channel without dangling bonds [138]. Jiyou Jin et al. demonstrated a dual gated coupled ReS_2_/h-BN/*α*-In_2_Se_3_ device structure who could function as high-performance non-volatile memory, programmable rectifier, and negative capacitance field-effect transistor and a minimum SS of 24.5 mV dec^−1^ with hysteresis free behavior when NCFET function had been spurred [139]. New kinds of 2D ferroelectric materials have been keeping on emerging in recent years thanks to the incessant endeavors dedicated by researchers, unfolding a new trajectory toward further improving the performance of 2D NCFET, providing monumental potential for 2D NCFET to be used commercially some day in the future [140].

##### Other Kinds of “Green” Transistors

**Fig. 5:**
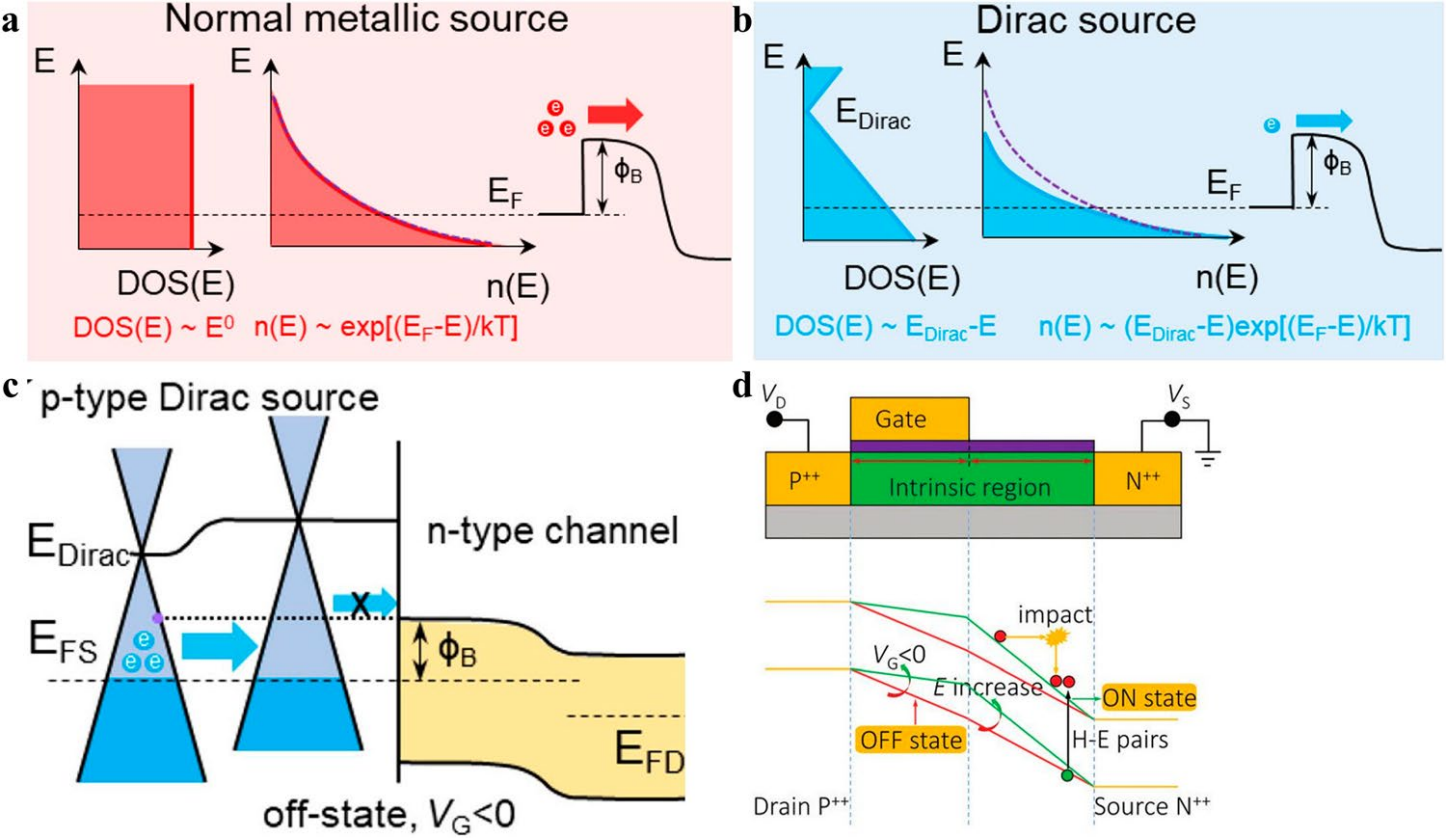
Energy vs density of states distributions of **a** normal and **b** Dirac source materials; **c** Band diagram of Graphene/MoS_2_ DSFET at off state [150]; **d** I-IFET device structure schematic and band diagrams for p-I-IFET at on/off state respectively [33]

Aside from 2D TFET and 2D NCFET that are extensively studied, other kinds of 2D materials-based transistors possess sub-thermionic SS encompass 2D DSFET, 2D S-FET or 2D I-IFET [38, 141, 142]. A 2D DSFET relies on Dirac source materials to filter out high-energy electrons to cut off the Boltzmann tail to obtain a sub-60 mV dec^−1^ SS, as showcased in Fig. 5a [143–145]. The sub-thermionic SS in DSFET arises from the DOS in Dirac materials decreasing rapidly with energy increasing, leading to *n*(*E*) super-exponentially decreasing with energy, thus suppressing the high energy thermal tail, which is a prevalent phenomenon in normal source materials whose DOSs are constant or increase in accordance with the increasing of energy, resulting in the electron density to decrease sub-exponentially or exponentially with the increasing of energy [143]. Graphene is the most widely studied Dirac source material, and it is hypothesized that gapped graphene can even hinder SS to a smaller value by virtue of the bandgap further hampering the high energy electrons distribution tail, leading to DOSs and *n*(*E*) to decrease more exponentially [144]. The on state current of DSFET is dependent on thermionic injection as depicted in Eq. (3) [143], where *q *is the charge carrier; h is Plank’s constant; *T*(*E*) describes the transmission probabilities from source to drain; *E*_Dirac_ denotes the energy of Dirac source material; Ф_B_ is the Schottky barrier that signifies the energy difference between the source Fermi level E_FS_ and the top of channel barrier. According to Eq. (3), SS can be smaller than 60 mV dec^−1^ when Ф_B_ < *E*_Dirac_, and attributed to the fact that the thermal injection of carriers determines the on state current, 2D DSFET can circumvent the roadblock of inferior I_on_ encountered by TFET,

3 $$\begin{gathered} I_{{{\text{therm}}}} = \frac{2q}{h}D_{0} \int_{{\Phi_{{\text{B}}} }}^{ + \infty } {dET(E)} \left| {E - E_{{{\text{Dirac}}}} } \right| \approx \frac{2q}{h}D_{0} \int_{{\Phi_{{\text{B}}} }}^{ + \infty } {dE\left| {E - E_{{{\text{Dirac}}}} } \right|} \exp (\frac{ - E}{{k_{B} T}}) \hfill \\ \begin{array}{*{20}c} {\Phi_{{\text{B}}} < E_{{{\text{Dirac}}}} } & {I_{{{\text{therm}}}} } \\ \end{array} = \frac{2q}{h}D_{0} k_{B} T\left[ {2k_{B} Te^{{\frac{{ - E_{{{\text{Dirac}}}} }}{{k_{B} T}}}} + (E_{{{\text{Dirac}}}} - \Phi_{{\text{B}}} - k_{B} T)e^{{\frac{{ - \Phi_{{\text{B}}} }}{{k_{B} T}}}} } \right] \hfill \\ SS = \frac{{k_{B} T}}{q}\frac{1}{{C_{1} }}\left[ {1 - \frac{{k_{B} T}}{{\left( {E_{{{\text{Dirac}}}} - \Phi_{{\text{B}}} } \right)}}} \right] \hfill \\ \end{gathered}$$

Ever since graphene has been used as Dirac source material in a 2D MoS_2_ based DSFET, and an average sub-thermionic SS of 40 mV dec^−1^ over four decades of current has been obtained with a large *I*_60_ of 40 μA μm^−1^, 2D DSFET has gone through a burgeoning and blossoming period of harvesting season, when prodigious kinds of Dirac/cold source materials have been investigated, and various kinds of novel source structures that function like cold source materials have been brought out [145, 146]. Apart from graphene, gapped graphene was consolidated to be capable of working as cold source as well by Fei Liu and coworkers. Furthermore, attributable to enhanced suppression behavior of gapped graphene over the thermal distribution tail brought about by the opened gap in graphene, a steeper switching behavior could be obtained in 2D DSFET based on gapped graphene Dirac source. An improved SS of 29 and 28 mV dec^−1^ had been attained in 2D DSFETs with graphene possessing gaps of 100 and 200 meV than that of 2D DSFET using gapless graphene Dirac source, whose SS was 57 mV dec^−1^. And intriguingly, the larger the bandgap in graphene, the smaller the SS was, which was accounted for by enhanced suppression ability over thermal distribution tail with larger bandgap [147]. With a comprehensive materials and device simulation model at atomic level, Juan Lyu et al. suggested that 2D transition-metal dichalcogenides and 2D transition-metal carbides were rich libraries for cold sources materials attributed to their specific density of states-energy relations. According to their simulation results, pristine graphene, doped graphene, Cd_3_C_2_, T-VTe_2_, H-VTe_2_, and H-TaTe_2_ cold sources based DSFETs with monolayer InSe as channel all could demonstrate SS below thermionic limit of 60 mV dec^−1^, serving as springboard for further studies to be conducted on low power consumption electronics [110]. Recently, 2D “cold” metal 2H MS_2_ (M = Nb, Ta) is speculated to be capable of achieving sub-60 mV dec^−1^ SS. Simulation results of cold metals-based MOSFETs with idealized structures demonstrated inspiring results that not only SS of such cold metals sources FETs (CM-FET) attained sub-60 mV dec^−1^ level, but also I_on_, cut off frequency *f*_*T*_ and negative differential resistance (NDR) also showcased superior results. NbS_2_/MoS_2_ CM-FET obtained a peak current of 4110 μA μm^−1^, several orders higher than the typical tunneling diodes owing to the broken bandgap structure of NbS_2_/MoS_2_ heterojunction. The largest peak-to-valley ratio of 1.1 × 10^6^ is obtained by TaS_2_/MoS_2_ CM-FET with V_GS_ of – 1 V at room temperature, providing practical applications for more Moore and more than Moore’s developing roadmaps [148]. Aside from 2D materials, 3D cold metals can also be fused with 2D channel materials to make 2D DSFETs that are feasible of making full use of the high density of states of 3D sources and superior electrostatic controllability of 2D channels. In 2023, Ligong Zhang and coworkers checked DSFETs with six kinds of 3D cold metals as sources, which includes three sulfides and three oxides, in combined with monolayer MoS_2_ as channel. Quantum transport modeling demonstrated that SS below 60 mV dec^−1^ spreading over five decades and on state current as high as 5.7 × 10^2^ μA μm^−1^ could be obtained in these 3D cold sources based MoS_2_ DSFETs, opening up new avenues for 2D DSFETs to boost I_on_ and offering a new territory of Dirac sources for DSFETs to fully delve into the performance potential of 2D materials-based DSFETs [149]. Other kinds of novel device structure as of GAA structure has been terrified to be capable of being combined with 2D DSFET structure to obtain steeper SS by virtue of Dirac source’s filtering effect and high *I*_on_ deriving from boosted gate controllability brought about by GAA structure [150]. What is more, theoretically, the 2D DSFET device structures are feasible of combining with other kinds of “green” transistor mechanism to fabricate 2D DS-NCFET [151], 2D DS-TFET and moreover, holding enormous potential to further minimize transistor dimensions with power consumption suppressed and sustain Moore’s law to even advanced technology node.

2D I-IFET makes full play of the famous avalanche breakdown phenomenon to achieve an abrupt off state to on state transition, resulting in an ultra-small SS. Concisely, avalanche breakdown depicts a phenomenon that an electron or hole with high kinetic energy originating from high electric field collides with lattice atom and impact its electrons or holes into free ones, and the kicked out electrons or holes further impact and ionize more free electrons or holes under the acceleration of an electric field, leading to an avalanche breakdown phenomenon and abrupt current multiplication. Attributed to the carrier multiplication effects and the abrupt and rapid function of electric field, I-IFET is characterized by high on state current and ultra-low SS, which have garnered monumental interests to I-IFET by virtue of its potential application in high performance low power consumption electronics [152]. The common structure of an I-IFET is a p-i-n or p-n diode structure, as demonstrated in Fig. 5d below, when *V*_*gs*_ < *V*_*t*_, the electric field is not strong enough to stimulate avalanche breakdown effect, thus the I_ds_ is dominated by the inverse drain current of the diode, which is limited to a small value, resulting in an ultra-low leakage current. As *V*_*gs*_ increase to above *V*_*t*_*,* avalanche breakdown phenomenon takes place abruptly and violently, leading to an ultra-low SS and high I_on_. Generally, in p-i-n I-IFET structure, the intrinsic region functions as a place where avalanche breakdown phenomenon takes place and tunes the length of the channel, eventually assisting in reducing bias voltage applied [142]. The 2D I-IFET combine the merits of high *I*_on_, small leakage and ultra-low SS possessed by I-IFET and the feasibility of reducing supply voltage required to spur carriers’ avalanche breakdown phenomenon, thus holding promising potential to renew ardors to 2D I-IFET to be used in low power consumption circuits [153]. Similar to 2D TFET, the fabrication process of 2D I-IFET also has been complicated by the asymmetric source/drain doping species, and what is more, the supply voltage of 2D I-IFET is high attributable to the fact that avalanche breakdown requires high electric field to ignite, contracting to the low power consumption purpose set by modern electronics, thus a plethora of endeavors have been devoted to decrement *V*_*gs*_ required in 2D I-IFET and promising achievements have been attained [154–156]. More attempts are needed to be dedicated to further diminish the supply voltage needed to trigger avalanche breakdown in 2D I-IFET to make it more appropriate to be used in modern low power consumption electronics.

##### 2D FinFET and 2D GAAFET

**Fig. 6:**
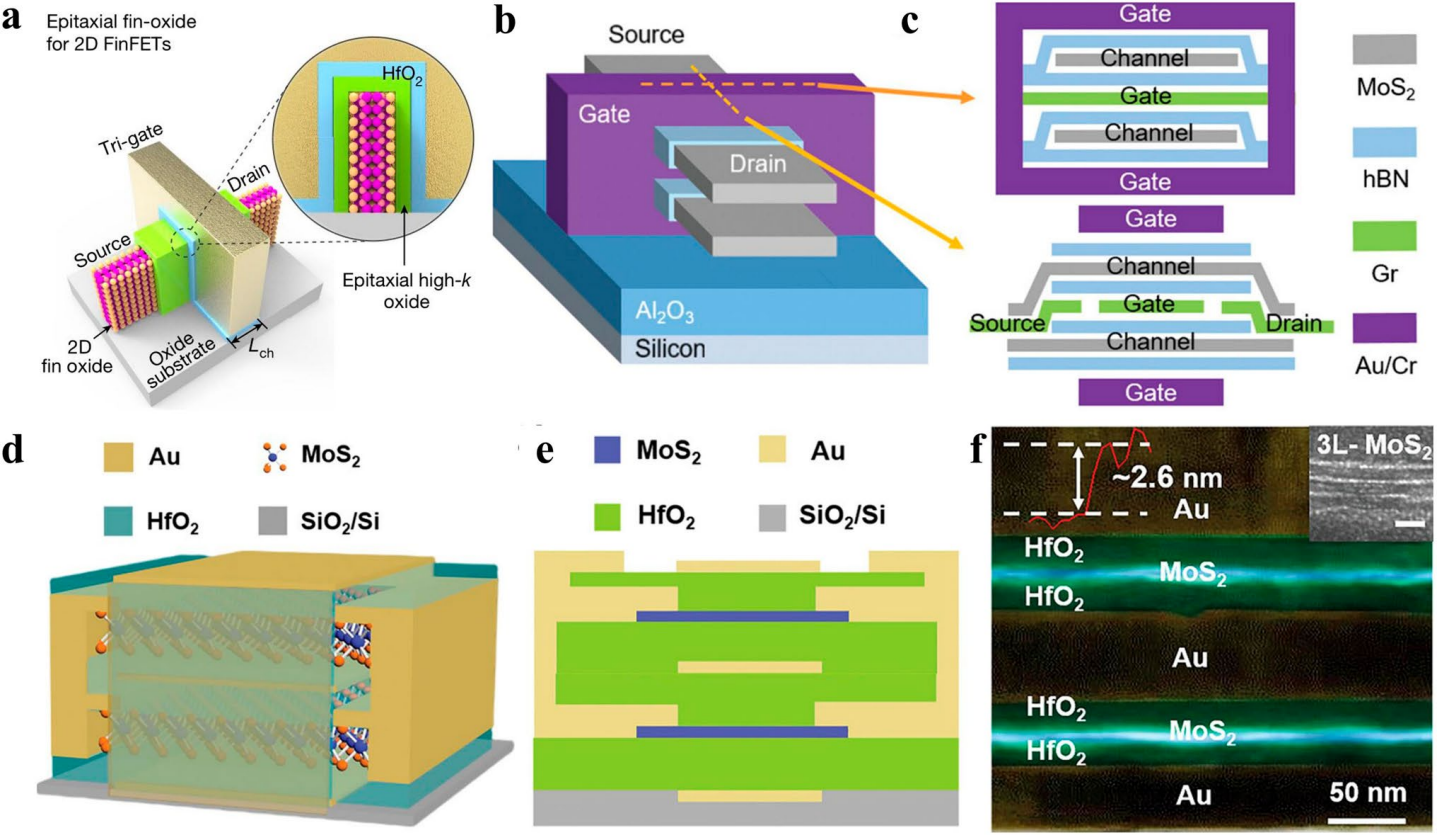
**a** A Bi_2_O_2_Se/Bi_2_SeO_5_ 2D FinFET device schematic [163]; **b** A 2-layer-stacked MoS_2_ MBCFET schematic and **c** the detailed profiles perpendicular to and along the channel directions [44]; **d** 3D schematic and **e** cross-section schematic of a MoS_2_ MBC-FET, **f** cross-section high-resolution transmission electron microscopy (HRTEM) image of MoS_2_ MBC-FET [168]

The incessant miniaturization of electronic transistors has hastened parturition of 3D transistor structures, encompassing the prevalent FinFET at present stage and GAAFET structure for the upcoming technology node. FinFET structure has been adopted to ameliorate the increasingly degraded SCEs and leakage currents coming along with the scaling down of channel length in planar transistor at 21 nm technology node [40]. Encouragingly and promisingly, the increment of gate number brought in by 3D FinFET structure has enhanced gate electrostatic controllability over channel, resulting in SCEs suppression and leakage current inhibition, guaranteeing the sustainability of Moore’s law further beyond 5 nm technology node [157]. The efficacy and achievements of adding gates number in suppressing SCEs and leakage current corroborated by FinFET structure have further incubated the GAAFET device structure, which is noted by its ultimate gate electrostatic controllability brought about by gates surrounding around the channel region. As one would expected, GAAFET structure has been proved to be more effective and testified itself to be feasible to further push the Moore’s law to sub-1 nm node [158]. As a matter of course, it is natural to combine 3D device structures with 2D semiconductor channels to realize the full functions of both their merits, that is, the superior gate electrostatic controllability coming along with 3D device structures and the SCEs immunity capabilities brought along by 2D semiconductor channels. In 2014, Kausik Majumdar et al. firstly checked the ballistic performance of an TMD-based FinFET structure with an ultimate gate length of sub-5 nm with simulation method, and found that an intact electrostatic integrity still remained in TMD based FinFET transistor at such limited gate length conditions, showing pronounced potential of 2D materials-based FinFET devices for ultra-low-leakage current applications with small footprint, excellent energy efficiency, and moderate performance [159]. The next year, Min-Cheng Chen and his coworkers demonstrated a 4 nm TMD-based FinFET with back gate control for the first time, showcasing TMD could potentially provide sub-1 nm thin monolayer body for 2 nm technology node [160]. Enormous endeavors have been dedicated to fabricate 2D materials-based FinFET devices that are compatible with modern semiconductor industry fabrication process. In 2019, Yu Pan et al. presented a MoS_2_ transistor with 10 nm gate length and p-doped Si fin as back gate electrode, which showed process compatibility with conventional Si-FinFET manufacturing process flow and marked the first time to realize large-scaled fabrication of arrayed MoS_2_ transistors. The MoS_2_ transistor comprised of a monolayer thin MoS_2_ channel of 0.7 nm, exhibiting super switching characteristics and large on/off ratio of over 10^6^, showing great promise of incorporating ultra-thin 2D materials into modern semiconductor industry [161]. In 2020, Mao-Lin Chen and coworkers fabricated a FinFET with one atomic layer fin, which was obtained by a template-growth method that fitted with different kinds of 2D crystals to isolate into monolayer in a vertical manner. The FinFET with one atomic layer thin fin presented an on/off ratios reaching 10^7^, shedding light on further diminishing fin width to atomic limit, holding promise to obtain electronics with larger integration density and lower power consumption [162]. In 2023, Hailing Peng and his research group members fabricated vertically aligned arrays of 2D Bi_2_O_2_Se/Bi_2_SeO_5_ epitaxial fin-oxide heterostructures, as demonstrated in Fig. 6a which demonstrated high electron mobility of 270 cm^2^ V^−1^ s^−1^, ultra-low leakage current of 1 pA μm^−1^, high on/off ratio up to 10^8^ and high on-state current of 830 μA μm^−1^ at 400 nm channel length, opening up new avenues for further extending Moore’s law. The native oxide of Bi_2_SeO_5_ had been obtained by layer by layer oxidation of 2D Bi_2_O_2_Se, which provided additional knob to obtain ultra-clean interface between 2D semiconductor channels and dielectric layers, paving avenues for eschewing the massive interface states commonly existing between 2D semiconductor channels and deposited 3D dielectric layers [163].

Aside from 2D materials-based FinFET structure, recently, 2D semiconductors have also been introduced into GAAFET (termed as 2D MBCFET) device structure to harness the ultimate gate electrostatic integrity of GAA gate structure and the SCEs immunity attributes of 2D materials. In 2018, Ruiping Zhou and Joerg Appenzeller demonstrated a 3D integrated multi-channel MoS_2_ FET device, the MoS_2_ channels were surrounded by double gates and the two-stacked MoS_2_ channels shared a common gate, which possessed the merits of super gate controllability brought in by GAA gate structure and the SCEs suppression capabilities coming along with the ultimate thickness of 2D semiconductor channels. Promisingly, the 2-stacked MoS_2_ device showed a high on state current of 535 µA μm^−1^, rivaling that of the recorded highest value, and an ultra-low leakage current of below 1 pA at V_ds_ of 1 V [164]. From then on, 2D material based multi-channel stacked device structures have witnessed burgeoning developments, and thriving achievements have been obtained, all of which have showcased high driving current and lower leakage current attributed to enhanced gate electrostatic integrity and ultra-thin channel thickness [44, 165, 166]. In 2021, Peng Zhou’s research group demonstrated a two-channel stacked MoS_2_ based GAAFET structure, the MoS_2_ channels both had thickness of 2 nm, and the normalized drive current of 23.11 µA*µm µm^−1^ in each channel exceeded that of 7-level-stacked Si MBCFET, with a ultra-low leakage current of only 0.4% of that of Si MBCFET, exhibiting the superiority of 2D channel materials in superseding Si in MBCFET, unfolding a new trajectory toward further expending Moore’s law [44]. In 2022, Hitesh et al. investigated a 3-level stacked MoS_2_ based MBCFET for the first time via simulation, where each channel was double-gated. The 3-level stacked MoS_2_ MBCFET showcased a high saturation current of 174.9 μA, with a near-ideal SS of 63 mV dec^−1^ and a high on/off ratio of over 10^8^, demonstrating a boosted current without compromising electrostatic control [167]. In the same year, Peng Zhou and Wenzhong Bao’s research group firstly fabricated large-scale MoS_2_-based 2-level stacked MBCFET by virtue of 2D materials’ merits of stackability, atomic thickness and marvelous electrical properties, the drive current of two-level stacked MoS_2_ MBCFET could reach up to 60 μA under 1 V bias, they also came to the conclusion that 2D MBCFET possess superior carrier mobility and SS, showing potential application in future semiconductor technology node [168]. In 2023, Xiong Xiong et al. fabricated 2-monolayer-MoS_2_-stacked GAAFETs on large scale, where GAAFET with channel length of 100 nm demonstrated *I*_on_ over 400 μA μm^−1^ per channel footprint at *V*_*ds*_ of 1 V and low contact resistance of 0.77 kΩ μm^−1^. The performance evolution trend was testified via checking hundreds of devices with various channel lengths, demonstrating the potential of monolithic integration of large-scale 2D materials thanks to their stackability and atomic thickness [169].

#### Memory Devices

Except for logic devices, the unremitting minimization of IC requires the memory region to be reduced as well. Memory devices are categorized into volatile and non-volatile types. Volatile memories are represented by dynamic random access memory (DRAM) and static random access memory (SRAM) who are characterized by high read and write speed while constrained by lower retention time that needs to be refreshed regularly by external power supply, resulting in a waste of energy. Non-volatile memories mainly comprises of flash, phase change memory (PCM), and ferroelectric resistor access memory (FRAM) who take the advantages of long retention time (longer than 10 years) even without power supply while suffer from lower data extracting speed, which limits their applications in computing process unit while can serve as movable disks that are indispensable in modern life. The relentless minimization of IC also set constraints and challenges for memory devices, that is, more dense, higher speed, longer retention time, and less power consumption concurrently. Thanks to their ultimate atomic thickness, 2D materials outperforms traditional 3D semiconductors in feasibility to reduce supply voltage to reduce power consumption effectively. On the other hand, the super stackability of 2D materials casts a new light on obtaining more inputs or outputs terminals in one single devices by leveraging the double surface of 2D materials, offering the opportunities to reduce device numbers in memory modules, thus reducing power consumption and footprint concurrently. The leakage current suppression ability of 2D materials ascribed to their ultimate thickness contributes to power consumption reduction. Thorough discussions of 2D materials-based volatile memories of DRAM, SRAM and typical non-volatile memory of flash have been comprehensively discussed below to give readers concise impression on 2D memory devices, and the non-volatile memories of PCM, FRAM which hinged on specific materials will be discussed in forthcoming work which specifies on memory device summary later.

##### 2D DRAM

DRAM is featured by a capacitor in combined with a transistor that contribute to footprint reduction, where the logic signal is stored in the capacitor, whose high speed of charging and discharging contributes to high read and write speed of DRAM while needs to be refreshed regularly by external supply power, resulting in power dissipation increment. As the semiconductor technology node evolves, the increasingly degraded SCEs brought about by device dimension down-scaling lead to leakage current increment, which is imperative to be addressed promptly by steering to new materials that are immunity to SCEs and capable of inhibiting leakage current. 2D materials are famed for their capabilities to counter SCEs and impeding leakage current attributed to their ultimate thickness, thus 2D materials-based DRAMs have engendered marvelous interest [170]. Among the many 2D materials, 2D TMDCs are widely considered as potential candidates to supersede or complement 3D Si in the future semiconductor industry, the ultra-low leakage current obtained in 2D TMDCs based MOSFETs has motivated the researchers to fuse them in DRAMs which mainly pay attention to transistor’s leakage current suppression ability. Chaitanya U. Kshirsagar et al. firstly fused a MoS_2_ 2D FET in a DRAM structure to leverage its ultra-low leakage current stemmed from the high bandgap of 1.8 eV in monolayer MoS_2_, which gave rise to leakage current as low as ~ 1 fA in DRAM circuits. For another, the retention time for 1 T/1C and 2 T gain cell memory devices reached as high as 0.25 and 1.3 s, showing dramatic potential in power consumption reduction [171]. Yin Wang et al. demonstrated a 2-transistor-1-capacitor (2 T-1C) configuration in 2021 based on monolayer MoS_2_ transistor and a metal–insulator-metal (MIM) capacitor, where 1 T-1C configuration functioned as a DRAM memory region while another transistor acted as the computing region. The 2 T-1C configuration demonstrated a multi-level voltage stages on the capacitor and long retention time thanks to the ultra-low leakage current of MoS_2_ transistor. They found that this 2 T-1C unit incorporating of computing and memory regions concurrently could function as a small in-memory computing circuit, offering potential opportunities to be integrated into future 3D dense lower power consumption circuits [172]. Using Monte Carlo simulation method, Mahdiye Raoofi and Morteza Gholipour compared a 2D TMDFET based DRAM with a Si-MOSFET based DRAM at the 16 nm technology node with fair conditions for different technologies, by evaluating the different performance of a digital circuits, they drew the conclusion that TMDFET-based DRAM consumed overall high power and demonstrated high variability than Si-MOSFET-based DRAM, whereas, TMDFET-based DRAM had higher timing characteristics [173]. In all, the overall researches about 2D DRAM manifested inspiring results, further endeavors are waited to be dedicated to the evaluation of their performance and limitations to further consider their value in lower power dense circuit application.

##### 2D SRAM

**Fig. 7:**
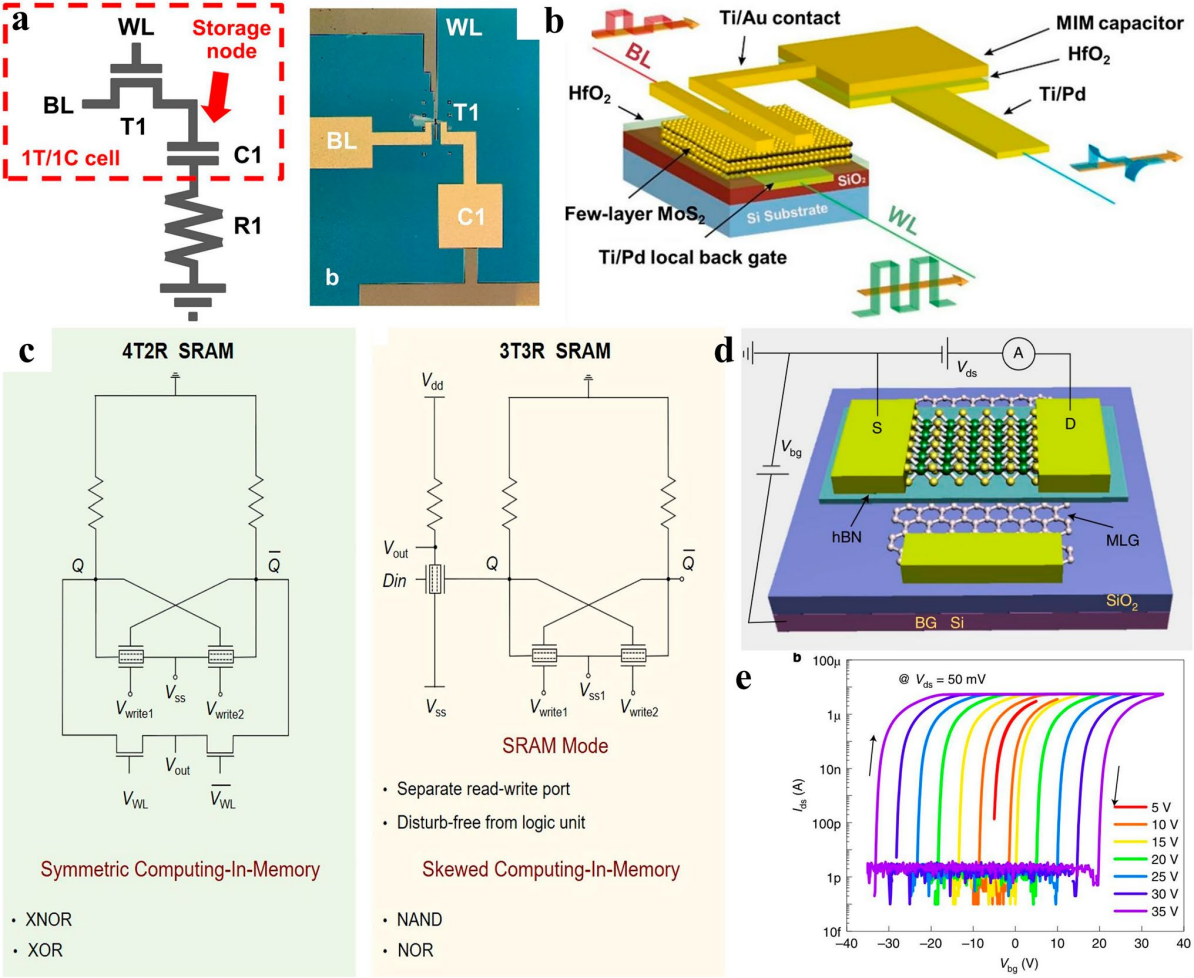
**a** Circuits schematic and Optical image of 1 transistor-1 capacitor (1 T-1C) DRAM made from MoS_2_ transistor and metal–insulator-metal (MIM) capacitor; **b** Illustration of 1 T-1C DRAM operation showing input and output signals [171]; **c** Symmetrical 4 transistor-2 resistor (4T2R) and skewed 3 transistor-3 resistor (3T3R) SRAMs computing-in-memory circuits with XNOR/XOR and NAND/NOR operations [177]; **d** 2D materials-based floating gate memory device with MLG as floating gate, MoS_2_ as channel, h-BN as tunneling layer, and **e** corresponding transfer characteristics of the device controlled by the back gate [180]

A typical SRAM comprises of 6 transistors, where four of them function as two cross-coupled inverters to store data while two of them act as access devices during the read and write procedures. Traditional SRAM based on Si transistors possesses a high speed arising from the high on/off ratio and large on-state current of transistors, the demerit of traditional semiconductor materials based SRAM is their large footprint attributed to unchangeable transistor numbers, leading to a large amount of power consumed. Attributed to their multi-input terminals of 2D materials-based transistors and ultra-low leakage current brought about by their ultimate thickness, 2D FET holds the promise of constituting SRAM with reduced transistor numbers, offering the opportunities to diminish power consumption and realize area-efficiency prominently. The first 2D SRAM was fabricated by exfoliated bi-layer MoS_2_ operating at D-mode (with negative *V*_*t*_) and E-mode (with positive *V*_*t*_), both of which showcased high on/off ratio over 10^7^ and high on-state current of exceeding 23 μA μm^−1^ at V_gs_ of 2 V and *V*_*ds*_ of 1 V attributed to high quality of bi-layer MoS_2_ brought about by mechanical exfoliation manner. The SRAM was constructed by a pair of cross-coupled inverters, which functioned as a stable memory cell [174]. A first WSe_2_-based CMOS SRAM was demonstrated in 2018, where a total of 6 transistors with chemically doped *p*-type 2D WSe_2_ transistor and electrostatically doped *n*-type WSe_2_ FET had been combined to construct a typical SRAM. By electrostatically controlling the doping level of the extended source/drain of the n-FET, noise margins could be suitably tuned to resolve the write/read conflict issues. As a consequence, the SRAM could operate stably at low voltage of 0.8 V [175]. Attributed to the two surfaces that can be used as input/output terminals, 2D materials provide the opportunities to decrease transistor numbers to attain the goal of reducing power consumption and footprint of SRAM without compromising its performance. For another, novel 2D SRAM structures keep on springing out attributed to a large abundance of 2D materials types. In 2019, Jiayi Li et al. fabricated a 2-transistor-2-resistor (2T2R) SRAM with 2D MoS_2_, whose two surfaces were both used as channels to augment input/output terminals, resulting in footprint reduction than conventional 6 T SRAM cell. The 2T2R SRAM exhibited stable read/write operations with 32.6%, 50.2% noise margins and 0.035, 0.036 μW power via optimizedly adjusting resistance and supply voltage conditions, showing potential in lower power consumption and area efficient chips applications [176]. In 2021, Fan Wang et al. demonstrated a symmetrical 4 transistor-2 resistor (4T2R) and skewed 3 transistor-3 resistor (3T3R) SRAMs based on two-surface-channels MoS_2_ transistor for in-memory-computing purpose, where the symmetrical 4T2R SRAM and skewed 3T3R SRAM were solidified to be capable of realizing in-memory XNOR/XOR and NAND/NOR computations respectively, as depicted in Fig. 7c. The two kinds of two-surface-channels MoS_2_ transistors-based SRAMs consumed less transistors than conventional typical 6 T SRAM, holding substantial promise in fabricating low power consumption, highly area-efficient and multifunctional computing chips [177]. The relentless development and plethora achievements achieved in 2D materials-based electronics also shed light on the evolution of 2D memory devices. Following the successful demonstration and efficacy in achieving area-efficient and lower consumption IC of 2D SRAM, in 2020, Guangyu Zhang’s research group realized large-scale 2D SRAM fabrication with a CVD grown 4-inch-scale monolayer MoS_2_, which demonstrated two stable operation states attributed to the superior performance of discrete MoS_2_ FET who showed ultra-high on/off ratio of 10^10^, high current density of ~ 35 μA μm^−1^ and high carrier mobility of 55 cm^2^ V^−1^ s^−1^ [178]. To evaluate the application potential of 2D SRAM, a simulation research performed by Yu-Cheng Lu et al. compared the 2D materials- and Si-FET-based SRAMs across various technology nodes across from 16 to 1 nm and verified that the 2D FET-based SRAM outperformed Si FET-based SRAM in terms of stability, operating speed and energy efficiency at all technology nodes checked. They used calibrated WSe_2_ p-FET and MoS_2_ n-FET to constitute the 2D FET-based SRAM. They found that at ultra-small technology node, take 1 nm as an example, 2D FET exhibited lower capacitance, leading to reductions in access time, write time and dynamic power by − 16%, − 60%, and − 72%, respectively, in cell. The obtained simulation results underscored the superiority of 2D materials-based transistors in ameliorating the performance degradation problems brought by dimensions downscaling of 3D semiconductors, showcasing the pronounced potential of 2D FET in application in more advanced technology nodes, offering an new avenue to fabricate high performance low power consumption IC chips [179].

##### 2D Flash

Flash memory, which is also termed as floating gate memory, is a common non-volatile memory, where the information storage is hinged on the transistor conductive characteristics variations tuned by an additional floating gate. Flash has a reputation for long retention time, high data storage capacity and superior data endurance, leading it to be prevalently used in movable data storage devices. A typical floating gate memory transistor is as depicted in Fig. 7d, which mainly encompasses a control/floating gates, a blocking/tunneling dielectric layer and a channel layer, where a floating gate lies between tunneling and blocking dielectrics and is charged or “erased” by the control gate. The flash memory store information depending on *V*_th_ shifting incurred by the applied control voltage. The working mechanism of a typical floating gate memory based on MoS_2_ channel/h-BN tunneling layer/multilayer graphene (MLG) floating gate layer/SiO_2_ blocking dielectric/Si control gate [180] is as following: at initial stage, a sufficiently negative voltage is applied on the Si control gate to “erase” the stored charges to make the memory to return to its original “− 1” state. Then a positive voltage is applied on the back Si control gate, when the positive voltage is sufficiently high, electrons in the MoS_2_ channel will tunnel into the floating gate, leading to the subthreshold voltage (*V*_th_) to rightly shift from state “− 1” to state “0”. Generally, the blocking dielectric is thick to contribute to a long retention time of charges in floating gate, namely, superior non-volatile characteristics. The tunneling dielectric should be optimizedly tuned to make it to be feasible of retarding reverse tunneling after the applied voltage has been removed to maintain the retention performance, whereas, it should not be too thick, otherwise insufficient tunneling takes place, which results in small programmable speed. Traditional flash has been encountered with scaling challenges nowadays as semiconductor industry enters into sub-5 nm technology node, where bulk Si channel suffers from promptly deteriorated carrier mobility attributed to dangling bonds scattering at ultra-thin thickness paradigm, leading to low operating speed. The degraded gate-coupling ratio and higher Si/SiO_2_ tunneling barrier contributing to shorter retention time and low extinction ratio. All of the limitations mentioned above constitute a pronounced hurdle for Si based floating gate memory to be used in low power consumption electronic circuits. The emerge of 2D materials-based flash memory sheds light on resurrecting the ardors to flash memory devices to ignite substantial attentions to be paid to them. 2D materials-based flash memory has gone through a long developing history. Simone Bertolazzi et al. demonstrated the first MoS_2_ based floating gate memory device with multilayer graphene (MLG) sandwiching between two HfO_2_ layers and monolayer MoS_2_ as channel. A 10^4^ difference between memory program and “erasing” states was attained thanks to the superior sensitivity of monolayer MoS_2_ to the charges in floating gate, paving the way for delving into the territory of 2D materials-based non-volatile memory devices [181]. In the same year, Min Sup Choi and coworkers fabricated all 2D materials-based floating gate transistors with large hysteresis that functioned as non-volatile memory devices. Graphene and MoS_2_ were used as channel and charge trapping floating gate with h-BN functioning as tunneling layer. They demonstrated controlled hysteresis and conductance polarities via changing the thickness or stack orders of the 2D materials, all of which manifested high mobility, stable retention, large on/off ratio and memory window, offering new avenues to fabricate flexible and transparent memory devices using atomically thin 2D materials [182]. MLG is favorably adopted as floating gate attributed to its high density of states independent of the layer numbers, additionally, MLG as floating gate can avoid the ion diffusion problems which are common in metal gates, suppressing leakage current and improving retention performance. Oxide materials also can been used as floating gate in floating gate memory device. In 2016, Qi Feng and coworkers demonstrated a non-volatile charge-trap memory device based on a few layer BP channel and a 3D Al_2_O_3_/HfO_2_/Al_2_O_3_ charge-trap gate stack. An unprecedented memory window exceeding 12 V had been attained deriving from the conspicuous charge trapping ability of high κ HfO_2_. The high program/”erase” current ratio, large memory window, stable retention and high on/off current ratio showcased the merits of combination of traditional high κ oxides with 2D materials to fabricate non-volatile memories, providing new avenues to build flexible, transparent and high performance 2D materials-based memory devices [183]. The researching goal of 2D materials-based flash device is to obtain higher operating speed, longer retention time, higher packing density, and multi-states that can contribute to lessen device counts for footprint reduction when constituting circuits, posing requirements for sharp interfaces with defect states suppressed, reasonably thick tunneling layers for charges to tunnel through with inverse tunneling suppressed, high capacitance blocking layer and sensitive channels that changes evidently with the presence of charges in floating gate. Nowadays, record low operating speed of 20 ns has been obtained in a MoS_2_/h-BN/MLG/h-BN/MLG all 2D flash and a MoS_2_/h-BN/MLG/SiO_2_/Si 2D flash, the all 2D materials-based flash showcased better endurance and retention performance than the Si/SiO_2_ based 2D flash memory attributed to ultra-sharp interfaces formed between MLG and h-BN layer, demonstrating the prodigious potential of 2D material based flash in application in low power consumption, high performance electronics [180, 184]. Reducing the supplied voltage applied on control gate to trigger erasing and writing of the floating gate by channel to comply with low power consumption purpose of modern semiconductor industry is another research focus of 2D materials-based flash devices, which has long been a monumental roadblock that plagues traditional flash which mainly comprises of high κ oxide or thick SiO_2_ as blocking or tunneling layers, posing rigorous requirements for driving voltage, whose downscaling lag has been dragged by the thick oxides materials. The straight forward avenue to reduce supply voltage is to diminish the thickness of blocking layer, whereas, the thinning down of blocking layer generally results in shortened retention time attributed to the possibility of breakdown of blocking layer or leakage current escalating caused by large supply voltage. Thus, retention time maintenance should reach compromise with supply voltage reduction contingent upon reducing blocking layer thickness [185]. With the development of 2D materials, 2D floating gate memory devices have been granted additionally enticing functions. Attributed to large memory window obtained, 2D materials-based floating gate memory devices possess the potential of storing multiple bits per memory cell, offering promising potential for ultra-high-density information storage [186]. A recently presented 2D floating gate memory device based on ultra-thin platinum disulfide (PtS_2_)/h-BN/MLG van der Waals heterojunction with atomically sharp interfaces showcased superior performance of ultrafast operations, a high on/off ratio (10^8^), extremely low operating voltage, robust endurance (10^5^ cycles) and retention time exceeding 10 years. Additionally, the reversible switch between positive photoconductive and negative photoconductive under different conditions could be achieved in the as fabricated 2D flash memory device, demonstrating the potential of fusing sensor-memory-computing [187]. In 2024, Thi Phuong Anh Bach and coworkers presented a 2D Te-based floating-gate device with excellent performance metrics of high switching on/off ratio (~ 10^6^), significant endurance (> 1000 cycles) and impressive retention (> 10^4^ s). Furthermore, the narrow bandgap of 2D Te endowed the floating gate device with broadband programmability from the visible to the near-infrared regions at room temperature. Moreover, multi-bits storage could be attained in the 2D Te-based floating gate device when different gate voltages, light wavelength and laser powers were applied, demonstrating the potential applications of 2D Te-based floating gate devices in optoelectronic memory [188].

2D materials-based floating gate memories not only showcase promising potentials to fabricate high density, low power consumption and high speed non-volatile memory devices, but also present promises to grant new functions to modern ICs thanks to their marvelous electronic and optoelectronic properties. Albeit enormous achievements have been attained on enhancing the speed of the memory, escalating packing density, and enabling multi-bits storage in a single memory device to make area efficient in 2D floating gate memory devices, the endurance and retention performance of 2D flash are generally inferior to that of the traditional floating gate memory devices. The main obstacle lies in that high quality and homogeneous 2D materials are lack at the moment. The defects in 2D materials which are unavoidable at the moment with the present synthesis techniques will finally contribute to the failure of the device, while the inhomogeneity of 2D materials will lead to device-to-device, and cycle-to-cycle variation, resulting in low yield, hindering their usage in circuit fabrication. The large contact resistance, immature transferring or fabricating techniques and inefficacy in pursuing van der Waals high κ blocking or tunneling layers integration, all these limitations pose monumental challenges for 2D flash memory devices to conquer. Fortunately, the destination is luciferous and full of wonders, which deserves substantial endeavors to be devoted to reach to.

### Electronics for More than Moore's

In the context of the persistent down-scaling trend of modern electronics, another More than Moore’s trend stepped under the spotlight except for Moore More’s development road for modern electronics. More than Moore’s refers to the concept that electronics of various functions are integrated into a same IC chip to obtain more functions to fulfill the diverse requirements raised by modern life. By optimizing the integrated circuit design and computing algorithm arrangements, more functional applications can be obtained in the same chip aside from diminishing electronics dimensions. The discrete electronics in the More than Moore’s regime mainly encompass memristor, memtransistor, sensors, flexible electronics and RF transistors. The recently prevalent memristor and memtransistor are pivotal in in-memory-computing (IMC), which dedicates to circumvent the “memory wall” (A concept that describes the computing time is shorter than the data shuttling time between memory and computing region, which results in energy and latency consumption waste.) issue which has been deteriorated to increasingly unbearable extent in von Neumann Architecture, or neuromorphic computing which fulfills the requirements of modern artificial intelligence computing [189]. Flexible transistors emerge as one of the core parts of modern ICs in the context that the wearable and fordable electronics come into the modern people’s life to offer more convenience. 2D materials-based sensors have garnered colossal attentions due to their large surface-to-volume ratio which are beneficial for higher sensitivity to light, gas or other analytes to be detected. With the advent of 5G or even 6G communication, RF transistors have ignited enormous interests with the increasing demand for greater bandwidth in communication systems, it is urgent that RF transistors can keep their operating frequency surging on pace with communication bandwidth evolution. 2D materials-based RF transistors offer new avenues to broaden the bandwidth attributed to their high carrier mobility and large drive current obtained with the scaling-down of transistor dimensions. Herein, the device physical mechanism and the current status of the devices mentioned above of More than Moore’s regime based on 2D materials will be discussed thoroughly in the manuscript below to give the readers an holistic concept of the more than Moore’s devices.

#### 2D Memristor and Memtransistors

**Fig. 8:**
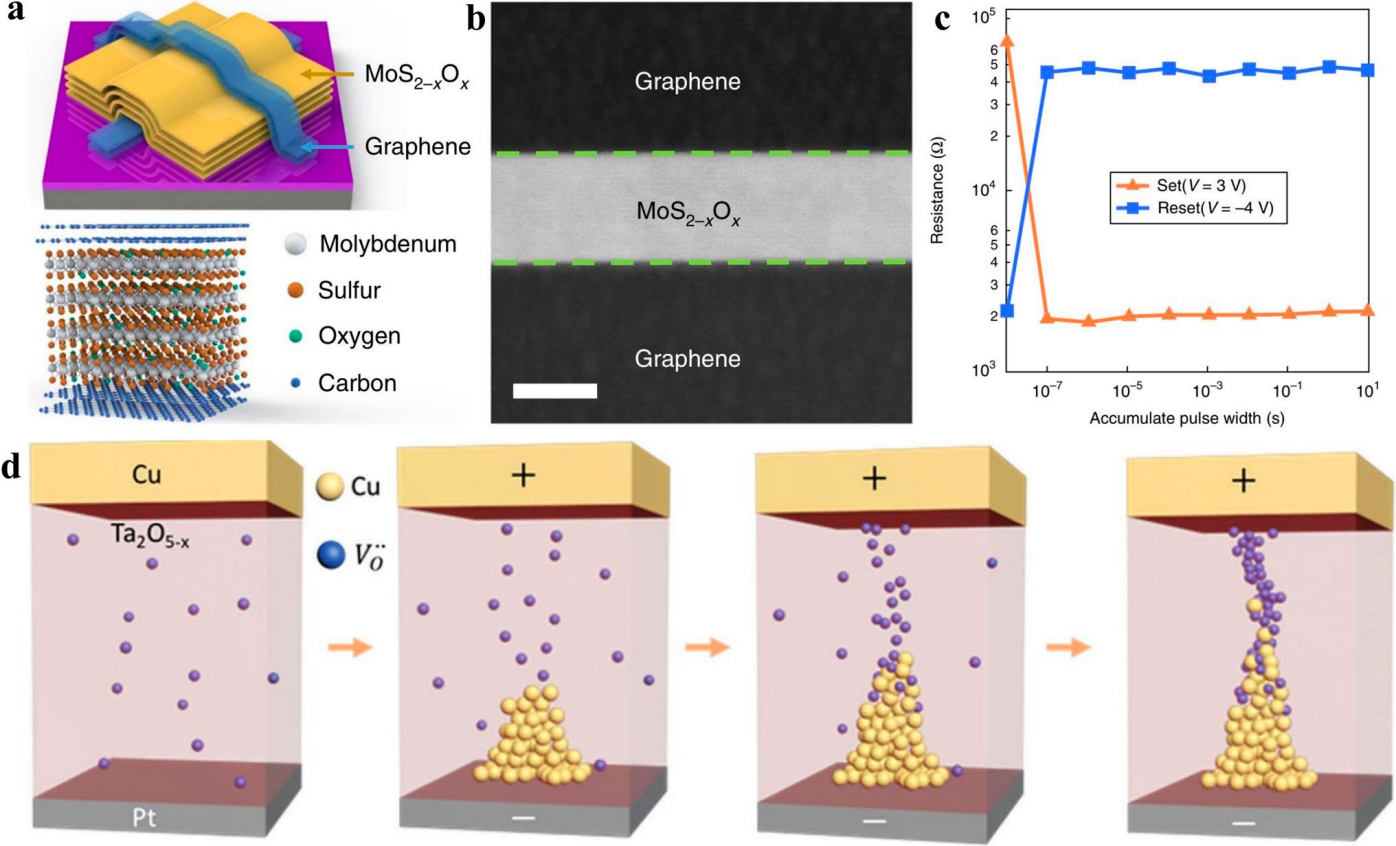
**a** Schematic of a 2D graphene/MoS_2-x_O_x_/graphene memristor. **b** Cross-section high angle annular dark-field (HAADF) image of a pristine graphene/MoS_2-x_O_x_/graphene memristor device and **c** its corresponding high and low resistance states [194]; **d** Schematic of filament formation of a memristor [199]

A memristor device generally comprises of three layer, a bottom/top electrode and a passive electric interlayer that possesses resistive switching behavior, as demonstrated in Fig. 8a below. The working mechanism of memristor is as below: initially, the interlayers are in high resistance state (HRS) due to their insulating or semiconducting behaviors, after applying a voltage between two electrodes, a current conductive path forms which substantially reduces the resistance of the interlayer, resulting in a low resistance state (LRS). Generally, the interlayer in traditional memristor is oxide, encompassing hafnium oxide, titanium oxide, tantalum oxide or phase change materials which can undergo numerous read/write cycles without damage, that is, ultra-high endurance and stability [190, 191]. Whereas, traditional memristors suffer from limitations that constrain their applications in modern IC chip. Memristors based on metal oxides are plagued by device-to-device or cycle-to-cycle variation issues which limit their applications in neuromorphic computing [192]. Memristors based on phase change materials encounter high power consumption brought about by large supply voltage needed for phase changing [193]. 2D materials-based memristors stand out and cast new light on neuromorphic computing and in-memory computing attributed to their lower power consumption deriving from their ultimate thickness, a large abundance of materials types that are feasible of fulfilling various kinds of requirements of modern integrated circuits [194]. For example, h-BN based memristor was proven to be capable of realizing small device-to-device and cycle-to-cycle variations, which might be due to the overwritten of defects by most conductive defects, making it less sensitive to defect formation during device fabrication [195]. The superiority of repeatibility of h-BN-based memristor offers it the opportunities to be fused into neuromorphic computing technology. Whereas, 2D TMDC-based memristor showcased larger device-to-device and cycle-to-cycle variations which might stem from the high defects states formed during device fabrications which contributed to stochastic conductive filaments formation [196]. Fortunately, these memristors with high working variations are popular in probabilistic systems, where processing variations are sources of randomness and are highly welcomed. For another, still much efforts have been devoted to reduce the working process variation problems of 2D TMDCs-based memristors attributed to the more mature device manufabrication process and large amounts of materials types, and promising improvements have been obtained [197]. The large abundance of materials types of 2D materials family results in different working mechanisms of 2D memristors, encompassing filaments formation, defect migration, phase change, photon response and ferroelectricity, among which filament formation and defect migration are most prevalent [198]. Figure 8d showcased a typical filament formation phenomenon in 2D memristor device, where the conductive components generally originate from the metal electrodes whose atoms penetrate into the interlayer under the supply voltage. Initially, the insulator layer without filament formation works in HRS without applied voltage, when a supply voltage high enough has been exerted on the electrode, the metal atoms from the electrode penetrate into the interlayer and form a conductive filament, which lead to an abrupt transition to LRS, whereas, when an sufficient inverse voltage is adopted, the formed conductive filament ruptures and the interlayer returns to HRS again [199]. Theoretically, conductive filament formation and rupture working mechanism in 2D memristor generally leads to relatively high process repeatability attributed to the priority on formed conductive path chosen by conductive metal elements from electrodes when the 2D materials have been optimizedly engineered. Defects migration, mainly comprising of vacancies and grain boundaries, is another 2D memristor working mechanism widely exist in 2D memristors. Attributed to their migration nature, the transition from HRS to LRS is not as abruptly as 2D memristors whose working mechanism is filament formation and rupture. As for the 2D materials with multiple phases, phase change is capable of acting as working mechanism in 2D materials-based memristors. Photosensitivity exists in particular kinds of 2D materials, take SnS, WSe_2_, and WS_2_ for example, where the phonic irradiation may cause large amounts of carrier emergence, leading to conductivity change in 2D materials, resulting in HRS and LRS in 2D materials-based memristors. The large abundance of 2D materials offer a thriving platform to fabricate 2D memristors for various purposes and fulfilling multifarious demands [200]. Aside from that, 2D materials’ ultimate thickness not only contributes to smaller supply voltage needed to save more power, but also results in enhanced gate controllability and SCEs immune properties in memtransistors, helping to fabricate low power consumption and high performance memristors or memtransistors.

#### 2D Flexible Electronics

Recently, modern life evolves into a scenario that cannot be imaginable formerly, when folded electronic book, prevalent robots, booming artificial intelligence, or bendable cellphone or notebook, all are enthralling, colorful and convenient, while on the other hand, posing more requirements for flexible ICs which are insurmountable to be fulfilled by traditional flexible electronic devices. As a result, researches on realizing the full function of flexible electronics have garnered massive attentions with the advent of fordable electronic devices, wearable or implantable electronics. Flexible electronics can be used as E-skins, wearable sensors, fordable and rollable displays and energy converters. Traditional flexible electronics are mainly based on organic materials since inorganic semiconductors as Si, Ge, or III-V semiconductors are brittle and easy to be broken up even though they are thinned down to very small thickness. Organic materials are subjected to instability and low device performance, and are incompatible with modern semiconductor manufabrication process. Recently, carbon nanotube, semiconductor nanowire, and thin-film ZnO are potential candidates for flexible electronics fabrications, while unfortunately, flexible electronics based on these materials suffer from low device yield and difficulties in fabricating large-scale flexible electronics [201, 202]. Fortunately, 2D materials have come to the rescue thanks to their ultimate atomic thickness, robust mechanicalness, fantastic scalability and superior stretchability, attributed to which, 2D materials can tolerate more deformation and withstand larger elastic strain than bulk semiconductors or organic materials [203]. Indeed, the Young’s modulus of most 2D materials are much larger than those of bulk semiconductors once normalized, taking graphene as an example, which has a much larger Young’s modulus (1000 GPa) than that of Si [204].

Transistors based on flexible substrates which encompass polyimide (PI), polyethylene terephthalate (PET) or Polydimethylsiloxane (PDMS) have been investigated for a long period of time. It is imperative that the flexible transistors’ performances are capable of being maintained notwithstanding the transistors have been bent to a certain extend. Traditional semiconductor materials are brittle and susceptible to failure after bending even to a small extent. 2D materials which possess marvellous mechanicalness and flexibility unfold a new trajectory to fabricate flexible transistors. By transferring to flexible PI substrate, Ick-Joon Park et al. fabricated a 2D MoS_2_-based flexible transistor with a tri-layers graphene as S/D electrodes, boding well for 2D materials-based mechanically stretchable transistors [205]. It is natural to hypothesize that all 2D materials-based flexible transistors are capable of withstanding stronger bending mechanical stress. To work out a credible conclusion, Saptarshi Das and coworkers demonstrated an all 2D materials-based flexible transistor where a monolayer graphene was used as electrodes, 3–4 layers thick h-BN was adopted as gate dielectric while a bilayer WSe_2_ functioned as a channel layer. The all 2D materials-based flexible transistor showcased promising performance, with a high mobility of 46 cm^2^ V^−1^ s^−1^, SS of 90 mV dec^−1^ and a high on/off ratio over 10^7^, well transcending that of the state-of-the art flexible transistor based on amorphous Si. For another, the all 2D materials-based transistor could sustain its performance under an in-plane mechanical strain of 2% [206]. The fruition obtained in 2D materials synthesis, transferring techniques and device performance boosting contribute to expediting 2D materials-based flexible electronics manufacturing on large scale. In 2020, Guangyu Zhang’s research group demonstrated large-scale monolayer MoS_2_-based transistors and circuits on a 4-inch-scale PI substrates, where the transistors were fabricated with high device densities (1,518 transistors per cm^2^) and high yield (97%), demonstrating high on/off ratio of 10^10^, high on-state currents of 35 μA μm^−1^, mobility of 55 cm^2^ V^−1^ s^−1^ and superior flexibility [178]. Transferring the patterned transistors structures to target substrates from sacrifice substrates has been terrified to be an effective avenue to reduce contact resistance in 2D materials-based transistors, inspired by which, Alwin Daus et al. had come up with a novel 2D materials-based flexible devices by transferring pre-patterned MoS_2_ transistor to a target flexible PI substrate, which not only contributed to maintaining the high device performance of MoS_2_ transistor, but also assisted in achieving ultra-short channel flexible transistors, the as fabricated MoS_2_ transistor channel length reduced from microscale down to 60 nm and an on-state current as high as 470 μA μm^−1^ had been obtained. For another, the transfer approach was suitable to other kinds of 2D materials apart from the prevalently adopted MoS_2_ [207].

Apart from 2D materials-based flexible transistors devices, other kinds of 2D materials-based flexible electronic devices comprise of 2D flexible photodetectors, sensors, displays or phototransistors, which are brought about to fulfill the versatile functions needed in modern life, for example, flexible displays used in fordable phone or paperwhite; flexible photodetectors or sensors for robot and e-skins. 2D flexible photodetector based on exfoliated 2D materials were demonstrated with marvelous performance, and the diverse and thriving 2D materials platforms offer the opportunities to fabricate flexible detectors capable of working in gamut wavelength [208]. By virtue of the achievements attained in synthesizing 2D materials with high quality in large scale, 2D materials-based flexible detectors arrays have found ways to come into views of flexible ICs. Domenico De Fazio et al. showcased a large-scale photodetector arrays that were manufactured by stacking centimeter-scale CVD graphene and centimeter-scale monolayer MoS_2_ together, which hinged on photo-excited electrons transferring from MoS_2_ to graphene channels, reaching an photo responsibility at least two orders higher than that made from bulk-semiconductor flexible membranes. The photodetectors demonstrated high photo-current under smaller voltage of 1 V, and were stable under a curvature upon bending to 1.4 cm, showing high potential in being applied in wearable electronics [209]. A all 2D materials-based flexible photodetector by vertically stacking semitransparent bis (trifluoromethanesulfonyl)-amide (TFSA)-doped graphene/MoS_2_/TFSA-doped graphene, which exhibited 0.128 A W^−1^ responsivity (*R*) and 1.69 × 10^9^ cm Hz^1/2^ W^−1^ detectivity at 532 nm wavelength with average visible transmittance being 58%. The 2D flexible photodetector exhibited excellent bending stability by remaining 32% of its initial responsibility after 2,000 bending cycles at a radius of bending curvature of 2 mm attributed to all 2D materials configuration, boding well for being applied in fordable and wearable electronics [210]. The self-healing capability and stable performance of flexible photodetector under a large deformation is a fundamental challenge for it to be used in wearable and fordable systems. Recently, Chunhua An and coworkers fabricated a flexible photodetector with self-healing capability and high performance stability after ongoing large deformation by adopting a self-healing 2D material which was made by homogeneously mixing 2D materials with self-healing polymer of imidazolium-based norbornene polymerized with ionic liquids and counterions. Under small tensile strain within 150%, the self-healing 2D flexible photodetector demonstrated stable or even increased photoresponse, which degraded when the tensile strain increased up to 1000%. Intriguingly, the as fabricated 2D flexible photodetector not only presented full recovery from repeated mechanical cuttings, but also demonstrated superior stability thanks to the stable additives, laying the groundwork for 2D flexible photodetector to be used in wearable electronics [211]. For another, the abundance of 2D materials also provides a platform for fabricating high performance flexible photodetector to detect versatile wavelength, from near-infrared to green or even blue light by appropriately adjusting the band diagram of heterostructures constituted by 2D materials [212]. Other kinds of flexible electronics based on 2D materials such as 2D flexible light emitting diodes for fordable display, 2D flexible sensors for wearable or artificial intelligence circuits have also witnessed booming advancements [213, 214]. Plethora efforts have been keeping on being engaged in the research paradigms of flexible transistors, detectors and related circuits, where leaps and bounds are awaiting to be made some day in the future [215–219]. Just as it is said, the destinations are full of wonders, blossoms and fruits, albeit to where the roads are fraught with thorns.

#### 2D Sensors

As modern life evolves, sensors have ignited colossal interests as the core part of the Internet of thing (IoT). Sensor is a kind of device that can detect the exact quantity of the specific kind of analytes to monitor its states. Generally, the analytes embrace dangerous or toxic gases, water molecular, protein and micro-organism and so forth. The recently developed sensor configurations comprise of surface-enhanced Raman scattering, plasma mass spectrometry, electrochemistry, chemiresistor, fluorescence, which are of high sensitivity while suffer from high cost fabrication, complex sample preparation and difficult operation [220]. Fortunately, sensor FET device configurations provide the merits of compatibility with modern semiconductor industry, low power consumption and high sensitivity, standing out and dominating the sensor paradigms. The sensors based on traditional semiconductor materials are constrained by their working conditions, such as high operating temperature (200 to 500 °C), variations in device performance, accuracy with humidity and long operating time [221]. Other potential candidates catering to fabricating sensor devices comprise of carbon nanotubes, semiconductor nanowires, and quantum dots, which possess high surface-to-volume ratios which are imperative to provide sufficient space for interacting with analytes detected. Albeit some promising results have been achieved, the 0D or 1D candidates face challenges either in large-scale sensors fabrication or device performance boosting. Recently, 2D materials have raised as stars in sensor electronics paradigm attributed to their high surface-to-volume ratio deriving from their ultimately atomic thickness which provides prodigious surface area for interactions between 2D materials with analytes. 2D materials-based sensors have gained prominent reputation thanks to their higher sensitivity, stability, repeatability, environmental friendliness and human environmental compatibility [222]. Generally, the 2D materials are hailed for sensitive surfaces which can be easily affected by the absorbed analytes, resulting in bandgap or conductivity changing, which can be easily captured by the variation in current drive-ability. The extremely large surface-to-volume ratio of 2D materials contributes to their ultra-sensitivity to a subtle change in the amount of the sensed analytes. 2D materials covers a large abundance of materials types, offering them a diverse platform for sensing assorted analytes, holding prominent potentials in being used as environmental or health monitor electronics. The rich surface chemistry of 2D materials and their conductivity susceptibility to external environments grand them to be superior candidates for sensor device fabrication. In 2007, Novoselov’s research group fabricated a first 2D graphene-based sensor for detecting gas which had beyond the reach of any resolution of existed detectors attributed to their high intrinsic noise. Graphene-based sensor was corroborated to be capable of detecting individual events when gas molecular attached to or detached from graphene’s surface due to its exceptionally low noise level. The graphene base sensor demonstrated a NO_2_ detection limit in the order of 1 ppb [223]. To enhance the detecting sensitivity of the 2D materials surfaces for specific analytes, sometimes, the surfaces of 2D materials have been treated with particular chemicals. In one research work, to reach the goal of detecting nitrogen dioxide, formaldehyde or benzene of small gas amounts and reducing reaction time, Marius Rodner et al. decorated graphene surface with iron oxide nanoparticles, attributed to which benzene and formaldehyde with concentrations as low as a single ppb of toxic volatile organic component (VOC) could be quantitatively measured and the response time had been reduced to less than a minute, which leading the sensor made from decorated graphene to be promising in air quality monitoring [224]. Apart from graphene, other kinds of 2D materials encompassing TMDCs, BP and metal oxides materials with finite bandgaps or metallic 2D materials are promising candidates for sensor FET fabrications deriving from their tunable bandgaps and conductivity. BP is hailed for its tunable direct bandgap and high carrier mobility, BP-based sensors have been intensively investigated and demonstrate promising results. A sensor field effect transistor based on multilayer BP had been demonstrated by Ahmad N. Abbas in 2015, which exhibited increased conductivity upon exposure to NO_2_ and showcased excellent sensitivity to NO_2_ down to 5 ppb [225]. While BP sensor possesses excellent sensitivity and electronic properties, its low environmental stability has exempted it from being treated as appropriate candidate for commercial usage. Recently, Zhenhua Liu et al. found that when In_2_O_3_ nanorods had been incorporated into multilayer BP to form hetero-structured BP-In_2_O_3_ composites to fabricated a sensor FET, which could demonstrate notably improved environmental stability and exhibit higher responsibility, lower response time, short recovering time and outstanding selectivity for NO_2_ detection [226]. 2D TMDCs are a large family group which comprises of various kinds of materials that possess different electronic or photonic properties, among which, MoS_2_ is the most prevalent and widely studied 2D material thanks to its suitable bandgap, environmental stability and relatively mature manufacturing techniques. MoS_2_-based sensors have been consolidated to be capable of not only sensing toxic gas such as NO_2_, NH_3_ [227, 228] but also detecting antibiotic with the assistance of DNA aptamer [229]. To improve the sensitivity of MoS_2_ based sensor to some specific analytes like NO_2_, illumination from light emitting diode with phonon energy matching with that of the bandgap of MoS_2_ was brought about by Tung Pham et al. to reduce the channel resistance of MoS_2_-based sensors, improved sensitivity to NO_2_ down to 4.9%/ppb had been obtained with Au electrodes, and further improved to 0.1 ppb when graphene electrodes coated with Au were used [230]. For another, a Pt doped MoS_2_ sensor which was prepared by hydrothermal method had been demonstrated with improved sensitivity, excellent repeatability and high selectivity, reaching a NO_2_ detecting limit of 0.1 ppb and a fast response/recovery time of 35/210 s to 32 ppm NO_2_ at 157 °C [231]. Except for surface modification by elements or chemicals, forming heterostructures with other kinds of materials offers additional knobs to render the sensors to be more sensitive and selective to specific elements needed to be monitored. A multilayer BP in combined with hollow ZnO hetero-structured (BP-ZnO) nanocomposites-based sensor demonstrated not only improved environmental stability which had been plaguing BP materials, but also enhanced the sensor’s responsibility, selectivity and achieved an ultra-low detection limit of 1 ppb for NO_2_ [232]. A 2D MoS_2_/Te heterostructure-based sensor exhibited enhanced sensitivity to VOC compared to MoS_2_ or Te sensors on their own. The sensor response was further enhanced under photo-illumination and zero-bias conditions up to values as high as 7,000% when exposed to butanone, providing a potential device architecture for portable and wearable sensor electronics [233]. Other kinds of 2D materials, modified 2D materials composites, and 2D materials heterostructures-based sensors have been keeping on emerging ascribed to the advancements attained in new materials pursuit, device fabrication and theoretical achievements. 2D materials-based sensors showcase promising application potentials in IoT and modern IC chip, whereas limitations and constraints such as lower detection limit, selectivity, instability, undesired contamination affecting performance of sensors lay aside immense hurdles for 2D sensors to culminate the ultimate goal of commercial application. Enormous efforts have been engaged in navigating the hurdles confronted by 2D sensors to make some headway, charting its course toward commercialization some day in the future.

## Integrated Circuits Based on Two-Dimensional Materials

Recent years have witnessed enormous achievements in materials synthesis, transfer techniques, device performance improvements approaches, and new materials or devices mechanism explosions in 2D materials regime. To culminate the ultimate goal of expanding Moore’s law by incorporating 2D materials superseding or complementing traditional Si semiconductor in modern semiconductor industry, it is indispensable to assess the capability of 2D materials in fabricating integrated circuits. Up to now, a plethora of 2D materials-based discrete devices have been reported, and promising results have been attained [234]. Whereas, attributed to the presence of defects in large scale 2D materials synthesized, which are susceptible to resulting in variability of figure of merits or parameters of devices, these variations may conduce to devices or circuits failure. Notwithstanding manifold low density ICs which are mostly fabricated from mechanically exfoliated 2D materials have been fabricated with promising results attained, large-scale integrated circuits with high density, such as microprocessor or memory arrays, are still sporadic. The fundamental reason lies in that large-scale 2D materials of electronic quality without defects that will lead devices or circuits to fail are still lacking hitherto, which set constraints to the scaling up of integrated circuits. Only numbered media-scale circuits based on 2D materials have been reported up till now, which put forward rigorous requirements on device yield and reliability, not alone large-scale integrated circuits with full functions, which are lacking at the moment [235]. Encouragingly, monumental efforts have been dedicated to the developments of 2D materials-based circuits, improvements have been accumulated in every small aspects of the paradigm, which will finally bring about disruptive breakthroughs some day in the future since as it goes, water constantly dripping wears holes in stone at last. Herein, a concise overview will be summarized to give a comprehensive view on the recent developing status of 2D materials-based IC.

### Small-Scale Integrated Circuits

#### Inverters and Logic Gates Circuits

**Fig. 9:**
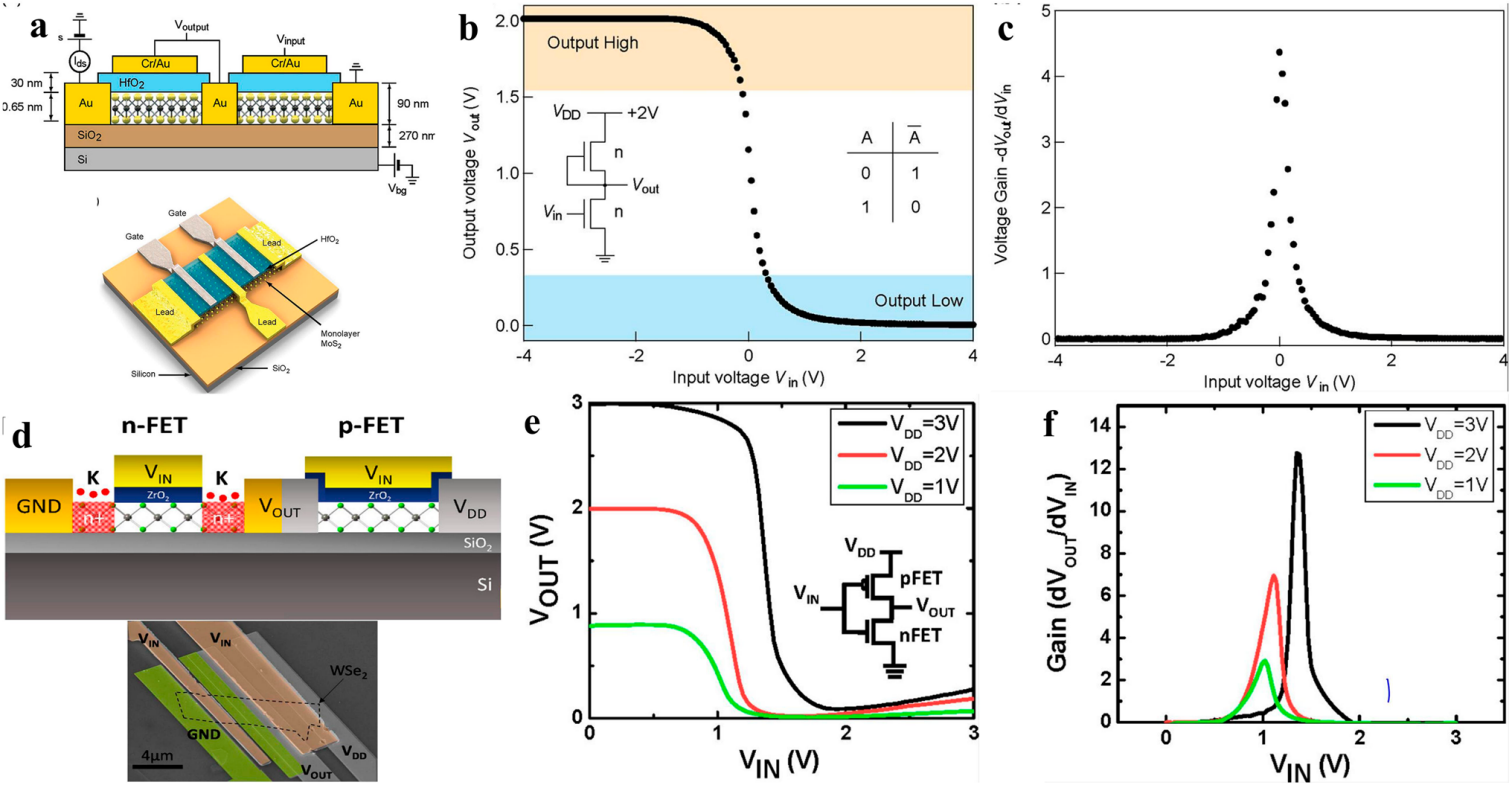
**a** Schematic structure of the first MoS_2_ based pseudo-inverter, **b** corresponding voltage transfer curve (VTC) (the inserting is the circuit diagram of the pseudo-inverter) and **c** voltage gain vs input voltage curve of the pseudo-inverter [236]. **d** Schematic and photo image of a CMOS inverter based on *n*- and *p*-type WSe_2_ transistor; **e** VTC and **f** corresponding voltage gain curves of CMOS inverter under different V_DD_ [238]

**Fig. 10:**
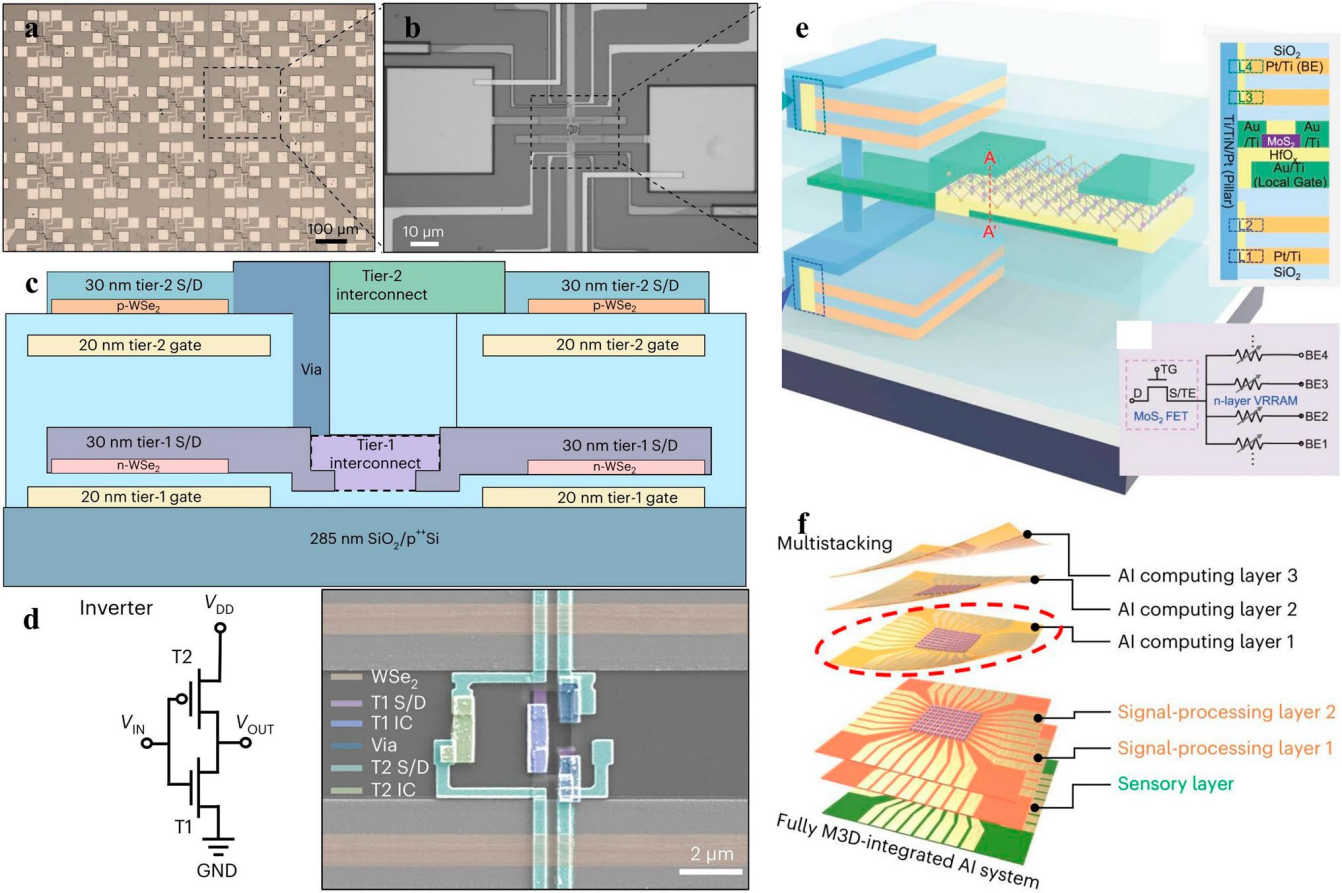
**a** Optical image of monolithic 3D integration of CMOS inverter based from tier1 and tier2, **b** Magnified scanning electron microscope (SEM) image of dotted region in **a**; **c** Schematic image of monolithic 3D integrated CMOS NAND circuits; **d** Inverter circuit diagram and false-color SEM image of relatively 3D integrated CMOS inverter [257]; **e** Schematic structure of monolithic 3D 1 T-4R structure and corresponding layer information, circuit diagram [255]; **f** Fully M3D-integrated AI nanosystem incorporating 6 layers, three computing layers, two signal processing layer and one sensory layer [256]

**Fig. 11:**
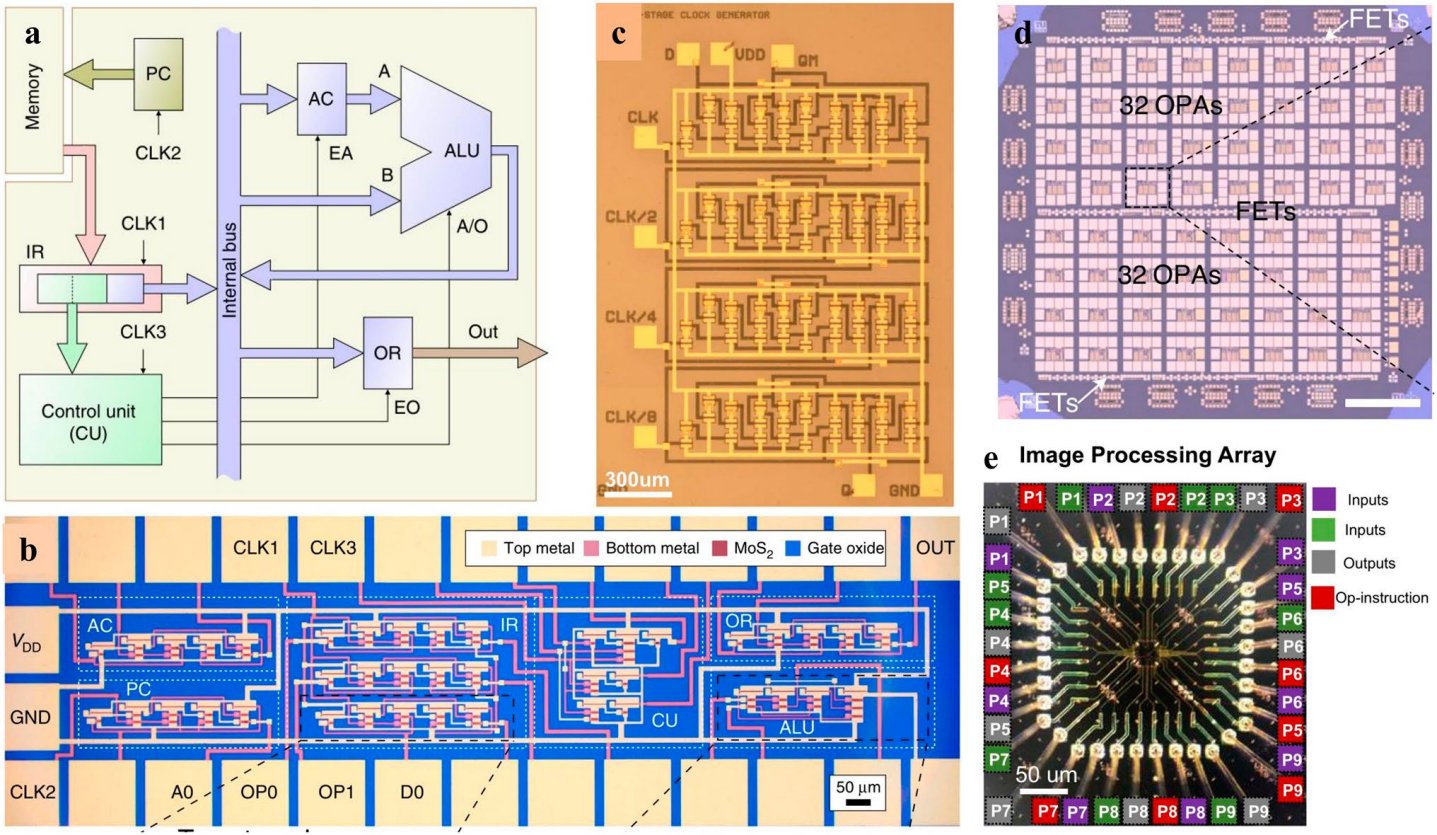
**a** Block diagram; **b** Microscope image of microprocessor [265]; **c** Optical image of MoS_2_-based clock division module fabricated on flexible substrate [270]; **d** Optical image of the hardware which contains 64 OPAs and test transistors [268]; **e** Optical image of the two-surface-channel WSe_2_ transistors-based image processing pix arrays, P1-P9 refers to the ports of pix units [267]

Inverters and logic gates circuits as NAND, AND, NOR, and OR are basic building blocks of logic circuits. Attributed to their relatively simple circuits diagrams, 2D materials-based circuits mainly focus on these small-scale circuits in the rudimentary stage, among which, 2D inverters, who contain only two components, generally a n-type transistor and a *p*-type transistor or a transistor combined with a load resistor, have garnered the most attentions and have been the circuits that was studied the earliest. It has been over 10 years since the first 2D material based inverter had been fabricated in 2011 by Branimir Radisavljevic et al., when they demonstrated a 2D material based integrated circuit that functioned as an inverter who could convert logic “1” to logic “0” and with voltage gain over 1, opening up an uncharted territory to steer to in the post Moore’s regime. The as-fabricated inverters were made from two n-type exfoliated monolayer MoS_2_ transistors, which was termed as “pseudo-inverter”, where one MoS_2_ transistor functioned as a resistor and another one worked as a switch by connecting the gate electrodes of the transistor acting as resistors with the drain of the switching transistor [236]. A high performance top-gated monolayer SnS_2_ transistor based on exfoliated SnS_2_ and corresponding logic gates of NOT and NOR with voltage gain of 3.5 and output swing > 90% had been demonstrated bySong et al., where the top-gated transistor structure allowed SnS_2_ to showcase a much improved mobility of ~ 50 cm^2^ V^−1^ s^−1^ than their back-gated counterpart whose mobility was ~ 1 cm^2^ V^−1^ s^−1^. This work paved the way for new candidates suitable for fabricating high performance 2D electronics and logic circuits [237]. For quite a long time, 2D materials-based inverters had adopted such configurations which were made from one kind of transistor due to the challenges confronted by doping in 2D materials and in lacking of *p*-type 2D transistors with matched performance. Inverters based on single kind of transistors suffers from higher power consumption attributed to large leakage current. What is more, it is hard for “pseudo-inverter” to achieve full-swing transfer curves deriving from leakage current flowing through the transistor who works as a resistor, thus resulting in a degraded voltage gain. A CMOS inverter can eschew the limitations mentioned above faced by pseudo-inverter by incorporating both *n*- and *p*- type transistors, which demonstrates no leakage current in ideal working conditions. The first CMOS inverter based on 2D materials was showcased by Mahmut Tosun and coworkers in 2014, when they fabricated a CMOS inverter by combining both a *n*- and *p*- type WSe_2_ transistors, where *p*-type WSe_2_ transistor was realized by adopting a high work function Pt contact metal to facilitate hole injection at the source contact and n-type WSe_2_ transistor was achieved by selective surface charge transfer doping with potassium to form heavily doped n^+^ contacts to contribute to electron injection. The CMOS inverter demonstrated an elevated voltage gain of over 12 under supply voltage of 3 V, where the relatively small voltage gain values stemmed from the inferior performance of n-type transistor in compared with that of *p*-type ones [238]. Except for WSe_2_, CMOS inverters based on other kinds of 2D materials as of BP, MoS_2_, or MoTe_2_ were also selectively doped via element doping, work function engineering, plasma-oxidized dielectric controlling, electrostatic doping or electron-charge transfer doping, resulting in high performance CMOS inverters on single 2D materials platform, paving the way for accelerating 2D materials-based circuits in commercial usage [239–242]. Most of the formerly reported 2D materials-based inverters were fabricated from mechanically exfoliated 2D materials, which set constraints to fabricate large-scale inverter arrays or relatively larger-scale integrated circuits. The logic gates such as NAND, NOR AND, and OR which occupied larger space were hard to be fabricated on exfoliated 2D materials attributed to their limited lateral sizes, which was lately addressed by 2D materials prepared by chemical vapor deposition (CVD), metal oxide chemical vapor deposition (MOCVD), atomic layer deposition (ALD) and solution processed method and so on. Thanks to the improvements made in preparing large-scale 2D materials with high quality, ReS_2_ transistors and logic gates arrays composing of NOT, NAND, NOR had been fabricated on CVD ReS_2_ and graphene electrodes, with an ion gel of high capacitance being used as gate dielectric, The ReS_2_ transistors allowed a below 2 V voltage to effectively gate the channel (Fig. 9, 10 and 11). The adopted CVD ReS_2_ and graphene opened up new avenues for large-scale ICs on flexible substrates [243]. Based on single ReS_2_ materials platform, Junyoung Kwon et al. demonstrated a NAND logic gate comprising a single transistor with graphene electrodes fabricated on atomically flat h-BN, which was selectively electrostatically doped by splitting gates, opening up a new gateway toward realizing “all-2D” logic gates on flexible substrates [244]. In 2018, Xiangfeng Duan’s research group adopted solution processing method to prepare large-scale 2D 2H-MoS_2_ with relatively high mobility of 10 cm^2^ V^−1^ s^−1^, based on which, large-scale electronics arrays encompassing transistors, inverters and logic gates composing of NAND, NOR, AND and even a half-adder had been fabricated, among which transistors showcased high on/off ratio over 10^6^. The reported large-scale 2D materials processed by solution method not only applied to MoS_2_, but also was capable of extending to fabricate other kinds of 2D materials, including WSe_2_, Bi_2_Se_3_, NbSe_2_, In_2_Se_3_, Sb_2_Te_3_, and even black phosphorus, demonstrating another pathway for fabricating large-scale 2D materials-based electronics and circuits [245]. Recent years have witnessed explosive developments in 2D materials-based inverters and logic circuits thanks to the achievements attained in high quality materials synthesis, increasingly mature and abundant transfer methods, and devices performance improvements [246–252]. Hyebin Lee and coworkers had ameliorated the transferring method of 2D materials and extended it to fully transfer patterned 2D transistors and circuits from sacrificial substrates to target flexible substrates, which was capable of perfectly reserving the performance of transistors and inverters, boding well for fabricating 2D circuits on arbitrary substrates [246]. Meng-Hsi Chuang et al. realized large-scale low static power consumption hetero-CMOS inverters based on n-typed MoS_2_ transistors and *p*-typed single-walled carbon nanotube based transistors. By tuning the logic geometry, a balanced inverter achieved high gain of ~ 67 under 2 V supply voltage, and what was more, an ultra-low standby power consumption of 5 pW was attained under supply voltage of 0.25 V with a practical peak gain of ~ 7, shedding light on fabricating wafer scale lower power consumption electronics [247]. To address the mobility mismatching issue existing in Si CMOS inverter between n- and *p*- type transistors, Wenzhong Bao and Peng Zhou’s research group demonstrated a heterogenerous CMOS inverter based on Si pFET made with silicon-on-insulator technology and *n*-type MoS_2_ transistor, where the mobility mismatching issue could be addressed by mobility matching and multiple-gates modulation of the MoS_2_. The well-tuned heterogenerous CMOS inverter exhibited a high voltage gain of 142.3 at a supply voltage of 3 V and a voltage gain of 1.2 with power consumption of 64 pW at a supply voltage of 0.1 V, which had been fabricated on a four-inch-scale substrate with high yield. The as-fabricated Si SOI-MoS_2_ CMOS inverter could function as a sensitive optoelectronic system that could convert the light signal into digital signal in one step, more efficient and cost effective than conventional light detecting optoelectronic systems. For another, they heralded that their SOI-MoS_2_ CMOS inverter could also function as effective gas sensor to detect NO_2_ and NH_3_ due to high sensitivity of both MoS_2_ and silicon to gases with oxidizing or reducing capabilities [248]. To add brilliance to the 2D materials-based inverters and logic gates’ present splendors, Xinran Wang’s research group reduced the parasitic capacitance of MoS_2_ transistor by incorporating an air gap, by depositing sub-stoichiometric AlO_x_ on MoS_2_ with ALD method, MoS_2_ transistor’s V_th_ could be tuned to be negative, which was termed as D-mode MoS_2_ transistor, whereas MoS_2_ transistor with positive V_th_ was denoted as E-mode MoS_2_ transistor. A D-E mode inverter based on two kinds of MoS_2_ transistors could be optimized to demonstrate a voltage gain of 9.2, switching threshold of 1.2 V and noise margin of 0.65 V, based on which a five stage ring oscillator (RO) had been demonstrated, which could successfully oscillated with operating frequencies reaching GHz regime, increasing by two orders of magnitudes compared with conventional back gated-based RO structures. The boosted working frequency was derived from the air gap introduced in the gate region, which contributed to parasitic capacitance dwindling [249]. Lun Dai’s research group demonstrated monolithic three-dimensional CMOS inverter arrays based on large-scale n-MoS_2_ and p-MoTe_2_ grown by CVD method, where n- and p- channel FETs stacked vertically and shared the same gate electrodes. The CMOS arrays exhibited high devices yield of 60% with an average voltage gain of 3.6 under a supply voltage of 1 V, among which the typical voltage gain and power consumption were 4.2 and 0.11 nW, respectively, under V_DD_ of 1 V. This research opened a new avenue to descend the footprint of CMOS inverter and obtain high density ICs [250]. Similarly, to reduce the footprint of CMOS inverter, Menggan Liu et al. fabricated large-scale ultra-thin channel nanosheet (NS) CFET based on CVD 1L MoS_2_ n-type NS FET and CVD 1L WSe_2_ *p*-type NS FET by stacking them vertically. Their statistical data of 22 CFETs demonstrated excellent uniformity, demonstrating a promising avenue to fabricate high performance, low power consumption circuits with high device density [251]. Xiaofu Wei et al. fabricated a pseudo-CMOS inverter based on a self-biased load transistor and n type MoS_2_ switch transistor, where a gapped channel forming a tunnable barrier, circumventing the polarity control of 2D materials and exhibiting a reverse-saturation current below 1 pA. Attributed to the self-biasing effect brought about by the gapped channel, the homojunction-loaded inverters demonstrated good rail-to-rail operation at a switching threshold voltage of around 0.5 V, with ultra-low static power of a few picowatts and high voltage gain of 241. Besides, other Boolean logic gates of XOR, AND, NAND, NOR, and OR gates had been demonstrated with pronounced performance [252].

3D monolithic integration has long been treated as the ultimate solution for the continued miniaturization of integrated circuits, especially for 2D materials which are hailed for superior stackability and scalability. In 2020, Guangyu Zhang’s research group demonstrated vertically integrated transistor via layer-by-layer technique, and the vertical integration of multilayered devices with different functions as memory, logic and sensors had been showcased as well, providing technological platform for high performance electronics with large density [253]. A CFET inverter can effectively reduce the footprint occupied by a common inverter whose n/pMOSs distribute horizontally, by virtue of vertically stacked n/pMOS in a 3D monolithic integration manner. By 3D integration of n-type MoS_2_ FET and p-type MoTe_2_ FET, Yin Xia et al. fabricated wafer scale CFET arrays with voltage gain of 7 V/V under supply voltage of 4 V. For another, logic gates circuits of NAND and NOR based on two vertically stacked MoS_2_ channels had been also presented successfully, demonstrating the potential of 3D integration in reducing footprint of ICs [168]. A monolayer MoS_2_-based GAA NSFET had been demonstrated by Fengben Xi and coworkers, where for the first time, gate first process in combined with critical point drying had been introduced to scale down GAA NSFET to channel width and length of 50 nm without damaging monolayer MoS_2_. The perfect conformal gate deposition on monolayer MoS_2_ was achieved by using trimethylaluminium (TMA) soaking treatment, resulting in the successful operation of monolayer MoS_2_-based GAA NSFET with on–off ratio over 10^6^, manifesting the superior compatibility of 2D materials with 3D integration process of modern semiconductor industry [254]. Maosong Xie et al. demonstrated a representative monolithic 3D integration of interleaved logic layer lying between two high density vertical resistive random access memory (VRRAM) layers to form one-transistor-four-VRRAM (1 T-4R) structure to minimize latency and energy consumption, where the interlayer with transistors could perfectly drive the two VRRAM layers into four resistance states. The whole fabrication process was integration friendly with processing temperature all below 300 °C, and the later fabrication process would not affect the performance of the former layers. The monolithic 3D structure demonstrated high density, high efficiency, fast operating speed and low power consumption, holding enormous potential for energy-efficient 3D on-chip memory systems [255]. Ji-Hoon Kang et al. demonstrated a 3D monolithic artificial intelligence (AI)-processing hardware by stacking six layers of transistors and memristor arrays made on bottom-up synthesized two-dimensional materials vertically. The monolithically stacked 3D AI nanosystem significantly reduced processing time, supply voltages, footprints and power consumption attributed to densely packed layers and dense interlayer connectivity, casting new light on fabricating unparalleled multifunctional computing hardware [256]. Rahul Pendurthi and coworkers demonstrated a monolithic 3D integrated complementary WSe_2_ FET, where n-type WSe_2_ FET were placed in tier1 and *p*-type WSe_2_ were placed in tier2. Tier1 and tier2 were connected by dense interconnecting vias of 300 nm with pitch < 1 um and vertically integrated logic gates encompassing 27 inverters, 12 NAND gates and 12 NOR gates had been demonstrated, highlighting the potential of 2D materials in fabricating densely packed 3D monolithic ICs [257]. Subir Ghosh et al. demonstrated monolithic 3D integration based on two tiers, where tier2 consisted of graphene-based chemisensors and tier1 encompassed MoS_2_ memtransistor-based programmable circuits, with more than 500 devices in each tier. The interconnect (I/O) layer was densely packed with an inter-via structures possessing density as high as 62,500 I/O per mm^2^, enabling the physical proximity between sensors and computing elements to be reduced to 50 nm, offering the opportunities to reduce latency in near-sensor computing applications [258]. Endeavors and attentions have been kept on dedicated to the developments of 2D materials-based electronics and circuits, breakthroughs and transformative achievements are waited to be broken out some day in the future once the time is right.

#### Memory Arrays for Computing

Except for widely investigated small scale logic circuits or inverters based on transistors, memory arrays that can fulfill some logic functions or realize in-memory-computing, in-sensory computing or neuromorphic computing have triggered enormous attentions recently. As mentioned in the former part, 2D materials-based DRAM or SRAM arrays are capable of performance logic functions via suitably designing the circuits [172, 177]. Attributed to their cross-bar arrays structures, 2D memristor possesses the merits of capable of shrinking down to ultra-mall dimensions without compromising performance, thus resulting in higher density device arrays than those constituted by transistors. Resistive memristor is characterized by HRS and LRS, which can be treated as two logic outputs, that is, HRS can be termed as logic “0”, and LRS can be denoted as logic “1”. As a rule of thumb, memristors can constitute logic gates to perform logic computing tasks. As it goes, the 16 Boolean logic gates can all be obtained by suitably choosing the input and output signal locations or types in RRAM, especially, the multi-states can contribute to more intricate logic circuits functions [259]. The memristors-based computing boasts merits of higher operating speed, data-intensive and capable of saving latency and energy. In 2020, Lei Yin et al. presented the first demonstration of logic functions of unipolar memristors based on 2D HfSe_2−*x*_O_*x*_ flakes and memtransistors based on MoS_2_/graphene/HfSe_2−*x*_O_*x*_. The HfSe_2−*x*_O_*x*_ memristor demonstrated superior performance with high switching ratio (> 10^6^), high operating temperature (106 °C), long-term endurance (> 10^4^ s), which offered it with high possibility for fabricating logic gates comprising of NOR and NOT, and displaying a D-type latch circuits. The memtransistor made from the HfSe_2-x_O_x_ memristor and a MoS_2_ transistor was capable of combining the superior memristive properties with a high performance transistor, offering wide tunability in electrical switching behavior, which could concurrently implement logic operation and data storage, successfully demonstrating a D-type flip-flop circuits, opening up new avenue for future in-memory computing [260]. After that, 2D materials-based memristor used in memory computing had incurred tremendous interests attributed to their superior performance, attenuate energy and latency consumption and significant tunability in fabricating various logic circuits [170]. Shakya Chakrabarti et al. realized logic locking for logic gates of NOT, AND, NAND, OR, and XOR gates with no additional overheads with MoS_2_ memtransistor, offering an efficient hardware security solution to subvert IC piracy and overbuilding [261]. Large-scale 2D memristors for neuromorphic computing had been conducted thanks to the advancements achieved in both device fabrication techniques optimization and large-scale 2D materials synthesis. Baoshan Tang et al. leveraged solution processed wafer-scale 2D MoS_2_ materials and fabricated large arrays of MoS_2_ memristors in wafer scale, which demonstrated marvelous endurance, long retention time, high on/off switching ratio with linear conductance updating characteristics. For another, a 3-layer convolutional neural network model had been presented to recognize National Institute of Standards and Technology (MNIST) handwritten digits with high recognition accuracy of 98.02% [262]. A PbSe_2_ based memristor demonstrated anomalous resistive switching behavior with two interchangeable reset modes: total reset and quasi-reset, leading it to be capable of emulating synaptic plasticity and implementing multipattern memorization using a cross-bar array architecture. Heterophase grain boundaries were formed by local phase transitions induced by electron beam irradiation, resulting in residual filaments along the grain boundaries, which could guide the formation of filaments [263]. By harnessing the two surfaces of 2D materials, a photoswitching logic circuits with basic logic functions of AND and OR in a single cell had been presented by Chunsen Liu and coworkers in 2020. For another, logic computing and data storage could be integrated into the same photo-switching logic cell by inserting an additional floating graphene gate, shedding light on constructing new chips that could convergent computing with storage for area-efficiency and new functions [264]. The abundance and richness of 2D materials have been keeping on bringing us new wonders and surprises and broadening our horizons, just as it goes, nothing is impossible when stepping onto the abundant and blossoming 2D materials platforms.

### Large-Scale 2D materials-based Circuits

Attributed to the lack of wafer scale 2D materials with electronic quality hitherto, large-scale 2D materials-based circuits are rare at the moment. A microprocessor based on 2D MoS_2_ were presented in 2017 by Stefan Wachter et al., where 115 transistors were integrated in a 1-bit microprocessor. The circuit comprised of all the basic building blocks that were common in microprocessors, thereby could execute user-defined programs stored in an external memory, perform logical computing and communicate with its periphery, constituting the most complicated circuitry at that moment [265]. For quite a long time, microprocessor based on 2D materials have been leveled off attributable to lacking of 2D materials complying with industry standards, since 2D materials synthesized from CVD method always present tremendous defects that will lead to device or circuit failure, imposing insurmountable challenges to further scale the 1-bit microprocessor up to multi-bits level. Albeit no pathbreaking breakthroughs have broken out during this period of time, promising achievements have been keeping on springing out relentlessly. In 2021, Wenzhong Bao and Peng Zhou’s research group made full use of machine-learning (ML) algorithm to evaluate the key process parameters that affected the electrical performance of MoS_2_ top-gated FET and by combining ML with grid searching, wafer level fabrication of MoS_2_ FET and circuits were guided to co-optimize the device performance. Wafer scale MoS_2_ FETs and a 4-bit adder employing industry-standard design flows and processes had been demonstrated, validating the application potential of ML-assisted fabrication optimization for 2D materials-based circuits [266]. In 2022, Senfeng Zeng et al. exhibited a pixel processing unit based on a single WSe_2_ transistor which could be switchable between AND and XNOR logic functions via drain voltage regulation on carrier injection barrier. The single transistor pixel processing unit was feasible to greatly reduce transistor consumption in logic circuits. The WSe_2_ pix unit integrated into an image processing arrays could perform different image tasks, which demonstrated < 16% transistors consumption compared with traditional theme at the same processing power, holding promise to remove the circuit redundancy issue in parallel computing [267]. Analogue circuits based on 2D MoS_2_ had been demonstrated recently by Dmitry K. Polyushkin et al. in 2020. A microchip with 64 operational amplifier (OPA), the basic building blocks of analogue circuits, and relative test transistors had been fabricated, where the OPA could operate stably with good performance. The OPAs’ use in feedback circuits including inverting amplifiers, integrators, log amplifiers and transimpedance amplifiers had been showcased, demonstrating superior compatibility with 2D materials platforms [268]. A lower power consumption flexible IC on large-scale had been showcased recently by Guangyu Zhang’s research group. Thanks to their newly developed high-quality monolayer (ML)-MoS_2_ wafers and the ultra-thin high κ dielectric/metal-gate technology, the large-scale flexible ICs with full functions could perfectly worked under supply voltage below 1 V, marking a key step forwards toward energy-efficient flexible IC fabrication [269]. A relatively transformative breakthrough in flexible circuit fabrication had been performed by Guangyu Zhang’s research group in 2024, which demonstrated a media-scaled IC on flexible substrate with 112 transistors thanks to the cooptimization of fabrication process leading to homogeneous large-scale high quality MoS_2_ material. Except for typical IC modules as NAND, XOR, half-adder and latch that were of high yield and homogeneity, a medial scale clock division module based on an edge-triggered Flip-Flop circuit was successfully demonstrated, showing promise development in 2D flexible ICs [270].

Attributable to the feasibility to scale down to nanometer regime and less area occupied, memristors are not as sensitive as transistors to 2D materials’ defects or grain sizes. 2D memristors or memtransistors based large-scale in-memory computing or neuromorphic computing circuits have witnessed more rapid developments than large-scale ICs based on 2D transistors. Guilherme Migliato Marega reported large-scale logic-in-memory devices and circuits based on floating gate field effect transistors based on large-scale MoS_2_ synthesized by MOCVD. Basic computing blocks of modern processors as half-adder or even three-input NAND had been demonstrated, the approach allowed to cascade different cells without intricate current–voltage conversion circuits, showing potential for reducing power consumption [271]. A two-kilobit non-volatile computing-in-memory macro made from three-dimensional vertical RRAM made with 55 nm CMOS process technique, which was consolidated to be capable of performing 3D vector–matrix multiplication operations with input, weight and output of 8, 9, 22 bits, respectively, and offered improved brain magnetic resonance imaging (MRI) edge detection and inference accuracies compared with conventional methods when tested using the Modified MNIST and Canadian Institute For Advanced Research (CIFAR-10) datasets [272]. In 2022, Akhil Dodda et al. reported an all-in-one hardware platform with 320 MoS_2_ memtransistors with low consumption energy, which was “self-sufficient” that included computing, storage, sensing and security. The “all-in-one” 8 × 8 arrays of crypto engines monotonically integrated with internet of thing edge sensors, which comprised of 5 memtransistors to accomplish sensing and encoding functions, offering approach to guarantee near-sensor security [273]. In the same year, a hybrid structure based on CMOS integrated circuits and h-BN cross-bar memristor arrays had been fabricated to implement extreme learning machine (ELM) algorithm, where the CMOS ICs were responsible for designing the encoder unit, while the h-BN cross-bar array memristors were used to implement the decoder functionality, demonstrating the compatibility of 2D electronics with modern Si CMOS platform [274]. A recirculated logic operation scheme using memristive hardwire and 2D materials-based transistor for cellular automata evolution had been proposed in 2023 by Yanming Liu and coworkers, significantly reducing hardware complexity and resulting in up to 79 fold reduction in cost compared to field-programmable gate array (FPGA) based approaches [275]. Akhil Dodda et al. demonstrated a 2D active pixel sensor (APS) based on monolayer MoS_2_ phototransistor arrays, where each pixel used one single programmable phototransistor which was capable of integrating image capture (sense) and image processing (compute) concurrently, resulting in significantly reduced footprint and energy consumption. The as-fabricated 2D APS arrays showcased near unity yield attributable to the homogeneity of monolayer MoS_2_ and mature fabrication techniques, shedding light on fabricating in-sensor processing circuits with energy and hardware burden reduced [276]. A heterogeneous non-von Neumann 2D CMOS platform was achieved by integrating large-scale n-type MoS_2_ and vanadium doped *p*-type WSe_2_ FETs with non-volatile and analog memory storage capabilities by Rahul Pendurthi et al. in 2022, on which inverters, a simplified 2-input-1-output multiplexers and neuromorphic computing primitives such as Gaussian, sigmoid, and tanh activation functions had been demonstrated, manifesting the feasibility of wafer-scale heterogeneous integration of different 2D materials to fabricate non-von Neumann 2D materials-based large-scale integrated circuits for the first time [277].

Ascribing to the mature synthesis methods for large-scale graphene of high quality, graphene based media or large-scale circuits have been reported to some extent, though the lacking of bandgap in graphene has been retarding it from being applied in logic transistors, its ultra-high electron mobility and environmental stability contribute to its being used in radio-frequency, communications, interconnects or photonic electronics [278]. In 2011, Yu-Ming Lin and coworkers fabricated a wafer-scale integrated circuit based on graphene on silicon carbide wafer, where graphene based field effect transistors and inductors were monolithically integrated to operate as a broadband radio-frequency mixer at frequencies up to 10 gigahertz, exhibiting outstanding thermal stability with little device performance degradation (less than 1 decibel) between 300 to 400 K, opening up new possibilities to achieve practical graphene technology with more functionalities [279]. Ascribed to its marvelous electron transport properties, graphene has been regarded as perfect candidate for fabricating millimeter-wave electronics. A monolithic integrated circuits made from epitaxial graphene with higher quality and enormously improved homogeneity had been presented which could operate at unprecedented frequency of 80–100 GHz. The as fabricated graphene demonstrated increased operating frequency and higher data achieving speed, elevating the graphene based RF circuits’ performance to be on par with that of the existing mature technology [280]. The inherent disorders associating with carrier transport in graphene FET synthesized by CVD method had been harnessed to constitute physically unclonable function (PUF), which was resilient to machine learning attack deriving from memristive properties of graphene and was showcased to be capable of operating with ultra-low power, scalable, stable over time, and reliable against variations both in temperature and supply voltage [281]. The unparalleled proliferation of data traffic in wireless communication regime in recent year ceaselessly imposes new requirements on capacity, which require electronics and circuits to be capable of operating at sub-THz frequency by 2035. A graphene based high speed sub-THz receivers was demonstrated by Soundarapandian et al. in 2024, which was terrified to be able to outperform the state-of-the art sub-THz receiver, offering a low cost, CMOS-compatible, small-footprint solution that could fulfill the size, weight and power consumption requirements for the upcoming 6G technologies [282]. Moreover, graphene can serve as marvelous channel material in ion-sensitive FET (ISFET) which is characterized by capability of transducing chemical composition variations information into electrical signals attributed to its large surface-to-volume ratio, extraordinarily high electron mobility, chemically inert basal plane and compatibility with covalent and non-covalent functionalization techniques that increase sensitivity and selectivity. In 2024, Andrew Pannone and coworkers highlighted the merits of incorporating machine learning to generate predictive models with extensive datasets obtained from ISFET sensor arrays based on graphene for classification and authentication tasks. Artificial neural networks that possess remarkable abilities had been trained with data generated from non-functional graphene ISFET sensor arrays to discern instances of food quality or safety concern, shedding light on revolutionizing subtle detection of environmental or chemical changing for wide spectrum of applications [283].

Neuromorphic computing based on 2D large-scale integrated circuits have witnessed impressive achievements attributed to their less aggressive requirements on quality, uniformity, and grains size of 2D materials. In 2019, a Gaussian synapses based on a heterostructures of atomically thin 2D MoS_2_ and phosphorus had been demonstrated by Amritanand Sebastian et al. for constituting probabilistic neural networks, which showed potential for resurrecting the quintessential scaling of computing by facilitating aggressive size scaling through 2D materials, offering energy scaling via Gaussian synapses and enabling complexity scaling by virtue of statistic neuron networks. Simulation results for classification of brainwaves using Gaussian synapses based probabilistic neural networks had been showcased, holding potential for fabricating high performance and low power consumption infrastructures [284]. Sarbashis Das and coworkers fabricated a biomimetic audiomorphic device that embraced the neurobiological architecture and computational map inside the auditory cortex of barn owl, which consisted of multiple split-gates with nanogaps on a 2D MoS_2_ channel connected to source/drain contacts for imitating the spatial map of coincidence detector neurons and tunable RC circuits for imitating the interaural time delay neurons, with global back-gating capability to demonstrate neuroplasticity to capture behavioral and/or adaptation related changes in the barn owl. Combined the virtue source model for current transport with finite element COMSOL multiphysics simulations, they found that the precision of biomimetic audiomorphic device surpassed the barn owl by orders of magnitude, demonstrating a new route for energy efficient neuromorphic computing [285]. In 2020, Thomas F. Schranghamer and coworkers demonstrated graphene-based multi-level (> 16) non-volatile memristive synapses with arbitrary programmable conductance states, which were hailed for desirable retention and programming endurance. Especially, the graphene based memristors enabled the weight assignments based on k-means clustering, offering greater computing efficiency over uniform weight quantization for vector matrix multiplication, which was essential component for any artificial neuron networks [286]. A nanoscale collision detector that mimicked the escape response of lobula giant movement detector (LGMD) neuron of locusts had been reported by Darsith Jayachandran et al., where the detector was fabricated from a monolayer MoS_2_ photodetector stacking on top of a non-volatile and programmable floating-gate memory. The escape response of large detectors arrays of 128,128× had been assessed by simulation method with the conclusion that the 2D arrays brought about additional benefit of resolving the directionality of collision, which was in-realizable in a single device. The as-fabricated collision detector consumed less energy and footprint, opening up new avenue for developing low-cost, energy- and area-efficient collision avoidance system for artificial intelligent vehicles [287]. In 2023, Subir Ghosh and coworkers fabricated an all 2D materials-based bio-inspired gustatory circuits for intimating physiology and psychology feeding behavior for the first time, where the graphene based chemitransistors served as artificial gustatory taste receptors that formed an electronic tongue, while monolayer MoS_2_ memtransistors constructed an electronic-gustatory-cortex comprising a hunger neuron, appetite neuron, and feeding circuit, benefiting for future emotional artificial intelligence [288]. In the same year, a bi-inspired visuotactile neuron based on the integration of a photosensitive monolayer MoS_2_ memtransistor and a triboelectric tactile sensor had been demonstrated, where the triboelectric tactile sensor was capable of capturing the essential features of multisensory integration. They also demonstrated a circuit that could encode visuotactile information into digital spiking events, where the probability of spiking was contingent upon the strength of the visual and tactile cues [289].

The evolution road of large-scale circuits based on 2D materials is rugged and rough, which is mainly constrained by the scarcity of large-scale 2D materials of electronic quality attributed to immature wafer scale synthesis methods. Whereas, strides have been made in all aspects, comprising of materials preparation, device structure optimization, device yield and stability elevating, and circuits algorithm refining, thanks to the abundant efforts devoted to 2D materials-based ICs both from academia and industry. The 2D materials-based ICs are in their infancy stages, when substantial challenges and massive room for improvement have been calling for more efforts and capitals to be devoted to the research paradigm. Albeit the road is laden with thistles and thorns, the mountaintop will be surmounted some day in the future.

## Challenges and Prospects

The persistent pursuit for higher speed, lower power consumption and lower cost ICs with various functions in post Moore’s regime entails the relentless miniaturization of electronic devices, which has been hampered by the seriously degraded carrier mobility in traditional 3D semiconductor materials. Table 1 summarizes the basic figure of merits of Si and 2D materials-based FETs, comprising of the *I*_on_/*I*_off_ ratios, carrier mobilities, device sizes, demonstrating the super SCEs immunity feasibilities of 2D materials in post Moore’s regime and the monumental challenges encountered by 2D electronics to achieve performances on par with those of Si-based electronics at the current stage. As the most promising potential candidates to supersede or complement Si in the post Moore’s era, 2D materials have been encountered with formidable and cumbersome challenges to be conquered to be applied in semiconductor industry some day in the future.

**Table 1:** Figure of merits of Si and 2D FETs extracted from literature

Materials	*L*_*g*_ (μm)	T_ch_ (nm)	*I*_*on*_*/I*_*off*_	*μ* (cm^2^ V^−1^ s^−1^)	SS (mV dec^−1^)	References
5 nm nFinFET	0.019	5	8.1 × 10^4^	1350	70	[290]
5 nm pFinFET	0.019	5	7.7 × 10^4^		65	[290]
MoS_2_		0.8	10^7^	70	150	[20]
Bi_2_O_2_Se			> 10^5^	300	75	[23]
MoS_2_	3.5	0.65	10^7^		70	[24]
MoS_2_	0.01	6	10^7^	3	120	[51]
MoTe_2_ ( n)	10	2.3	10^6^	8.9		[70]
BP	1	5	10^5^	280	190	[77]
InSe		32	10^7^	3700		[89]
WS_2_		4.2		112		[97]
SnS_2_	3.7	1.8	10^7^	50		[237]

Fundamentally, wafer-scale synthesis of 2D materials with electronic quality that are homogeneous, with low defects densities, having large grains size and high mobility are imperative. Mechanical exfoliation has been consolidated to be an effective way to provide 2D materials with high quality, based on which, most state-of-the-art electronic devices that have set records in some figure of merits have been reported. Unfortunately, 2D materials made from mechanical exfoliation methods suffer from small lateral sizes, uncontrollable thickness and low yield, which lead mechanical exfoliation to be impractical for large-scale electronic integrated circuits fabrication. Notwithstanding various kinds of 2D materials can be synthesized from CVD method massively, enormous improvements are required to fulfill the stringent demands lay out by industry applications, among which defects reduction, grain sizes increments and materials uniformity escalating are dominant parameters that retard CVD from being capable of preparing industry standard 2D materials to be used in 2D materials-based VLSI circuits at the moments. Nevertheless, CVD methods are cost-effective, which contribute to unremitting interests being garnered both from industry and academia to ceaselessly improve the quality of 2D materials grown by CVD method and promising achievements have been attained thanks to the ameliorations in equipments, growing process and precursors selections. The state-of-the-art medium-scale ICs based on flexible substrates demonstrated by Guangyu Zhang’s research group in 2024 was made from monolayer MoS_2_ grown by improved CVD method, boding well for large-scale low cost 2D integrated circuits fabrication [270]. That is to say, the quality of 2D materials is the decisive parameter that determine the quality and even success or failure of the 2D ICs. MOCVD has recently emerged as a promising synthesis approach for 2D materials with large-scale and high quality, and increasing amounts of 2D materials with high quality have been prepared by this method regardless of its higher cost. Solution method is also feasible to prepare large-scale 2D materials with low cost and high yield, only that 2D materials prepared by this method are susceptible to suffering from lower carrier mobility and large defects densities, impeding their usage in fabricating large-scale 2D ICs. For another, the bandgap and electronic properties of 2D materials are dependent on their thickness, the large scale, high quality and thickness controllable synthesis methods for most of the 2D materials for being applied in different electronic device structures are lack at the moment. Since as it goes, one can’t make bricks without straws, thus synthesizing large-scale 2D materials that are capable of fulfilling industry standards is imperative for 2D materials capable of being fused into modern integrated circuits.

2D materials-based electronics are proposed to be capable of effectively conquering SCEs. Unfortunately, performance of 2D materials-based electronic device seldom outpaces that of state-of-the art Si transistor, especially in ultra-scale technology node ascribed to the non-optimized 2D electronic device structures, leading to unrealized performance potential of 2D materials-based electronics. Contact engineering, dielectric integration and precisely doping of 2D materials are the main paradigms to devote efforts to improve the performance of 2D materials-based electronic devices.

The contact resistances of 2D transistors are generally several orders of magnitude higher than those of Si transistors, which mainly originates from the difficulty in degenerately doping of 2D materials. Reducing the contact resistance in 2D materials-based electronic devices has long been a monumental obstacle that has plagued the researchers since the contact resistance, acting as the throat of the carriers injected from drain to channel regions, determines the drain current to a large extent. To reduce the contact resistance in 2D materials-based electronics, one feasible avenue is to adopt edge contact instead of top contact, where edge contact eschews the van der Waals gap which will increase the carrier tunneling barrier, leading to reduction of contact resistance. Whereas, edge contact is daunting to be realized in experiment, one compromise procedure is to steer to hybrid contact structure, where edge contact and top contact coexist to reduce the contact resistance concurrently. Deriving from the dedicate surface of 2D materials, traditional metal deposition method often results in serious damage to 2D materials, where Fermi level pinning caused by metal induced gap states (MIGS) generally exists, which will suppress the work function tunability of contact metals, resulting in a large contact resistance. Adopting 2D metal electrode such as graphene or ameliorating the Fermi level pinning effect by inserting h-BN, oxide layers such as TiO_2_ and Al_2_O_3_ to reduce the contact resistance have been extensively studied [97, 291, 292]. Semi-metal as Bi or Sb was demonstrated to be capable of forming ohmic contact with monolayer MoS_2_ due to the suppression of MIGSs in semi-metals contributing to suppression of Fermi level pinning effect, leading to substantially reduced contact resistance of 123 Ω μm^−1^ and quantum limit ohmic contact of 42 Ω μm^−1^ in Bi and Sb contacted MoS_2_ transistors respectively [293, 294]. Phase engineering is another effective method to reduce contact resistance in 2D electronics owe to the various phases for 2D TMDCs, where metallic phases can be adopted in source/drain regions by phase engineering to reduce contact resistance with electrodes [72]. MoS_2_ doping by Yttrium to transfer it into metallic to form ohmic contact with 3D metal was presented in 2024 by Jianfeng Jiang et al., achieving an average of contact resistances of 69  Ω μm^−1^ and total resistances of 235  Ω μm^−1^ with 10 nm-channel-length MoS_2_ transistor fabricated on a two-inch wafer, opening up new avenues to reduce contact resistance in 2D transistors on large scale [295]. Thanks to the achievements obtained in transfer of metal electrodes, van der Waals contact with transferred electrodes has reduced the contact resistance substantially attributable to the integrity of 2D materials without damage to the crystals of 2D materials, shedding light on wafer scale integration of van der Waals contact on 2D transistors [296, 297]. The former contact engineering efforts have dominantly been devoted to *n*-type transistors, especially MoS_2_ transistor who has been the researching star in the 2D materials-based electronics or circuits by virtue of its mature fabrication process and environmental stability. Milestones obtained in diminishing contact resistance in *p*-type 2D transistor are rare attributed to higher hole injection Shotty Barrier (SB) height arising from Fermi level pinning effect. Drawing on reducing contact resistance by degenerate doping to reduce SB width in Si transistor, Mayukh Das and coworkers had developed a high performance *p*-type MoSe_2_ transistor via degenerately substitutional p doping with Nb, in combining with a layer-by-layer thinning method to obtain thin channels region while leaving contact regions thick, an impressive benchmarking contact resistance of 95 Ω/µm had been achieved with high I_on_ of 1.8 mA μm^−1^, indicating the potential in improving the performance of *p*-type 2D transistors [298]. Fortunately, improvements are going on and accumulating for both *n*- and *p*-type 2D FETs, disruptive breakthroughs are waiting somewhere in the front, which marks the headway that the contact resistance in 2D electronics can be comparable or even outpace that of Si-based electronics, that is to say, the contact resistance of 2D electronics is feasible of fulfilling the standards set by the IRDS for the post Moore’s technology nodes.

Dielectric engineering is another roadblock lays in front of the 2D electronics in their commercial application road, which is derived from enormous trap states existing in the interface between 2D channel and 3D dielectrics. The incumbent gate dielectric materials in Si dominant industry is HfO_2_, which is formidable to be deposited on 2D channels ascribing to the absence of dangling bonds on 2D channels, resulting in islands formation at thinner HfO_2_ thickness and leading to inscalability of dielectric materials. Besides, the interfaces between 2D channels and 3D dielectrics are fraught with interface states, which are susceptible to resulting in larger leakage current, device failure, variations and instability issues, preventing 2D materials-based transistors from realizing their full potentials of performance. 2D h-BN with high environmental stability and relatively large bandgap (5.9 eV) attests to be capable of forming van der interfaces between dielectrics and 2D channels, impeding the interface states that seriously degrade device performance. Nevertheless, the permittivity of h-BN is low (ε ~ 5), leading to larger tunneling leakage current at ultra-thin thickness, exempting it from obtaining sub-1 nm EOT [93]. Native oxides of some 2D materials such as HfS_2_, Bi_2_O_2_Se, which could be oxidized into quasi-2D HfO_2_ or 2D dielectric Bi_2_SeO_5_ with high dielectric permittivity and ultra-clean van der Waals interfaces with 2D channels prove to be effective dielectric materials in 2D transistors. Unfortunately, this means are limited to several specific 2D materials [100, 299]. Forming quasi van der Waals or van der Waals interfaces between gate dielectrics and 2D channels prove to be effective avenues to successfully integrate gate dielectrics into 2D transistors. Hitherto, epitaxial CaF_2_, atomically thin c-Al_2_O_3_ grown by ALD, MnAl_2_S_4_ multilayer flakes, single-crystalline GdOCl, atomically thin MgNb_2_O_6_ crystals, and even perovskite SrTiO_3_ have been demonstrated to be feasible to form van der Waals or quasi van der Waals interfaces between 2D channels with interface trap states being suppressed, among which atomically thin c-Al_2_O_3_ prepared by ALD method, epitaxial MgNb_2_O_6_, CVD grown GdOCl all demonstrated higher κ values, large-scale integration feasibility, presenting promising usage in commercialized 2D electronics [24, 300–304]. In another work, the freestanding nanomembranes of perovskite SrTiO_3_ synthesized using hybrid molecular beam evaporation (MBE) were substantiated to possess incipient ferroelectricity at room temperature and showcase stable ferroelectric order below 100 K, which contributed to its capability to fabricate robust cryogenic memory devices at low temperature and construct physical reservoirs for pattern recognition at room temperature, paving the way for fusing 3D perovskite materials with marvelous properties into 2D materials-based electronics, enabling the integration of diverse materials into silicon complementary metal–oxide–semiconductor technology [305]. For another, wafer-scale transfer of 3D dielectric patterns from sacrifice substrates directly to target substrates with pre-patterned 2D electronics has been realized, paving a new promising avenue to integrate high κ dielectric materials with 2D channels with interface trap states inhibited, which is compatible with incumbent semiconductor manufacturing process as well [306]. Endeavors have been persistently devoted to the “right” 2D dielectric materials pursuit and newly efficient and facile techniques to integrated prevalent high κ oxides into 2D transistors with interface states impeded, abundant achievements have been attained up to now, thus it is hopeful in the future, dielectric integration issues in 2D electronic devices can be perfectly addressed and the roadblock be gotten around successfully.

It is impractical to dope 2D materials precisely alike traditional 3D semiconductors with high energy ion implantation attributed to the fact that the atomically thin thickness and delicate crystal structures of 2D materials are subjected to damage. To fabricate CMOS inverters which are cornerstones of modern integrated circuits, both n- and p- type 2D semiconductors are indispensable, and what is more, one kind of material platform for CMOS inverter fabrication offers the opportunities to substantially simplify the fabrication process instead of adopting different kinds of 2D materials, thus it is imperative to find avenues to precisely doping 2D materials with carrier conductivity types definitely controllable. There are some feasible avenues to effectively doping 2D materials have been reported. Chemical doping can effectively dope 2D materials to be *n*- or *p*-types according to the chemical reagents adopted, whereas, chemical doping generally proves to be unstable, which results in device performance degradation or device failure. On the other hand, the chemical doping processes are often associated with toxic organic solvents, which may cause damage to 2D materials and introduce residues or defects that will give rise to performance degradation. Electrostatic doping is verified to be an effective technique to dope most 2D semiconductor materials, applying additional voltage may enhance, deplete or change the conductivity properties of 2D materials, leading to selectively doping in 2D materials. Unfortunately, electrostatic doping often requires additional electrodes, which will complicate the fabrication process of 2D electronic devices, unfavorable for electronic device dimensional scaling down. The passivated oxide materials Al_2_O_3_ have been proved to be capable of negatively doping MoS_2_ transistor, leading its subthreshold voltage to be more negative and resulting in high on state current [307]. NH_3_ or N_2_ plasma spurred by ultra-low power were demonstrated to be able to dope 2D materials by substitution doping techniques, whereas, vacancies, trap states and damages could be introduced during the doping process, resulting in huge difficulty to precisely control the doping concentrations and the quality of 2D materials [308]. Introducing donor or acceptor elements during the 2D materials synthesis process can result in well controlled, stable doping in 2D materials via perfectly engineered process, though defects such as vacancies or trap states may be introduced in the synthesis process, the doping density and materials quality can be optimized by adopting appropriate doping species and perfectly adjusting the grown process. A recently published CMOS inverter fabricated from precise doping of 2H-MoTe_2_ to *n*- and *p*- type with controlled carrier concentration with Re and Nb substitutional doping, the doped 2H-MoTe_2_ demonstrated high quality by delicately tuning the synthesis process, CMOS inverter arrays based on as grown *n*- and *p*-type MoTe_2_ presented a voltage gain of 38.2 accompanied by a peak static power consumption of 89.5 nW at *V*_*dd*_ of 4 V, casting new light on precisely doping 2D materials to both *n*- and *p*-types in large-scale [309]. In all, the practical doping techniques that apply to large scale precisely controlled doping of 2D materials at the moment is via substitution doping during the synthesis process, which are capable of obtaining high quality 2D materials with accurately tuned doping types and concentrations in large scale.

Attributed to the hurdles in synthesizing large-scale homogeneous 2D materials with electronic quality, wafer-scale transferring procedure, challenges in fabricating 2D electronic devices mentioned above, 2D materials-based electronics suffer from large device-to-device and cycle-to-cycle variations issues, which results in the instability of the devices, posing significant challenge to fabricate 2D materials-based circuits, especially the VLSI ones that encompass tens of thousands of devices, which has not been realized up to now. The variation issues of 2D electronics are fundamentally dominated by wafer-scale synthesis of 2D materials. The present large-scale 2D materials synthesized via CVD, MOCVD, MBE, ALD, or liquid methods generally possess large amounts of defects, including vacancies, interstitials, grain boundaries, cracks or multilayer island regions, which are ineluctable at the moment with present growth techniques, resulting in notorious device variations and low yields problems that lay aside insurmountable obstacles to fabricate pure 2D materials-based VLSIs since the failure or misfunctions of the devices may lead to false signals in the circuits, which finally accumulates to circuits failure as well. Furthermore, wafer-scale precisely transferring of 2D materials from sacrificial growth substrates to target substrates without damages or residues has long been a bottleneck that shields 2D materials-based circuits from upgrading. The commonly adopted wet transfer process generally results in polymer residues or surface damage by noxious solvent, while the lately developed dry transfer method suffers from limited materials size, and moreover, cracks may emerge during the transfer process. Other kinds of transfer techniques including wet-dry mixed transfer process, metal assisted transfer or wafer bonding have been developed, all of which prove to be promising but are unable to obtain clean and intact 2D materials like the as-synthesized ones at the present stage. The defects and cracks introduced during transferring process also contribute to device variation issues. Moreover, there exist environmental, equipment difference and even the different states of the persons who perform the experiments, all of which will lead to device-to-device and cycle-to-cycle variations of 2D electronic devices ascribed to the ultra-sensitivity of 2D materials to external conditions. The fundamental factors that determine the device variations in 2D electronics are still the 2D material quality, other external factors mentioned above can be ameliorated by performing the experiments in more stable environments or referring to automatic machines to perform the fabrication process. Device variations will keep on existing until someday breakthroughs in fabricating large-scale uniform 2D materials of high quality come into view.

In all, the 2D electronic devices with performance optimized contingent not only upon high quality 2D materials synthesized with required doping types, concentration and optimized device structures, but also hinge on reduced contact resistance to boost on state current, perfectly integrated high κ gate dielectric to obtain enhanced electrostatic controllability to suppress SCEs and leakage current. Reducing the 2D electronic devices variations by referring to uniform and high quality 2D materials also should deserve equal attentions being paid on as those to improve discrete device performance, since uniform electronic devices arrays with high yield, performance stability and low variations are indispensable and imperative for further fabrication of 2D materials-based ICs.

In the circuit level, it is natural to hold the view that the successful function of 2D materials-based circuits rely on every discrete electronics in the circuits to function properly and work at their best states, and even one small device’s failure will conduce to the misfunctions of the ICs. Thus, to prepare 2D materials-based integrated circuits, homogeneous 2D materials of electronic quality, device performance being optimized to their best states, high devices yield that has better to draw on unite, high device endurance and low variations, high stability are necessary to make sure that the ICs can work properly apart from circuit design optimization. The first 2D materials-based 1-bit processor was reported in 2017, no substantive progresses in this paradigm have been demonstrated since then, the reasons lie in that disruptive achievements are yet to be attained in 2D materials synthesis with industry standards; on the other hand, most modern 2D ICs are based on only *n*- or *p*-type device that is fabricated from one kind of 2D material to simplify the manufacturing process, which impedes the optimization of the integrated circuits and set even high requirements to design the circuits; thirdly, device yields, stability, endurance and variations are about to fulfill the rigorous requirements set by an properly functioned integrated circuits. All in all, there is a long and harsh way to go to get 2D materials-based ICs to outpace or even be comparable with those based on state-of-the-art Si electronics.

Integrating 2D materials with modern Si dominated semiconductor fabrication process is of momentous importance for 2D materials-based electronics or circuits to be commercially used some day in the future. Whereas, the process is confronted with massive challenges and obstacles. The modern Si manufabrication processes involve high energy bombarding or high temperature annealing process which may lead to noteworthy damage to 2D materials, resulting in device failure or performance degradation, thus new doping techniques for 2D materials that are modest and compatible with Si production lines are desperate to be developed to integrated 2D materials into modern Si based semiconductor industry. Additionally, when 2D materials are chosen to be channel materials in superseding Si, the high κ metal gate (HKMG) integration process may encounter challenges as well, where the direct deposition of high κ HfO_2_ on 2D channel often results in substantial interface states stemming from absence of nucleation sites caused by dangling-bonds free surface of 2D materials. The plethora interface states will lead to gate leakage current increase, and what is more, dielectric breakdown may happens once the interface states accumulate to exceed critical value, leading to device failure. Adopting van der Waal high κ materials or van der Waal high κ in combined with 3D high κ oxides stack to form sharp interfaces with 2D channels with interface states suppressed proves to be effective avenues to integrated HKMG onto 2D channels, whereas, this procedure involves 2D materials transfer process, which is also critical and difficult to be performed in practical Si production line. To fuse 2D material into Si production line, wafer-scale transferring process automatically with precise alignment and no damage to 2D materials are imperative, whereas which are impractical at the moment. An alternative option is to grow 2D material directly on below substrate, which proves to be perfect since transferring process more or less will lead to residues, small cracks or winkles in 2D materials, resulting in performance degradation or yield decrement. Nonetheless, growing 2D materials of high quality always involves high temperature annealing, which may deteriorate the following process, thus, low temperature growth of 2D materials with high quality on arbitrary substrates are needed to fulfill the requirements of protecting the as fabricated infrastructures below and high quality channel materials to realize high device performance concurrently. At the present stage, it is impractical to totally replace Si by 2D materials in semiconductor industry, one favorable option is to integrated 2D materials-based electronics with Si based circuits to attain additional functions. In these context, obstacles that should be conquered encompass precise transferring process and effective void contact formation with below Si circuits.

Fortunately, hopes and opportunities exist anyhow and successes are yet to come someday in the future. Though it is impractical to completely supersede conventional 3D semiconductor with 2D materials to fabricate integrated circuits at the moment, 2D materials have found their place where they can fulfill their potential by complementing or even replacing some traditional semiconductor materials in specific regimes. Attributed to their high carrier mobility, environmental stability and superior mechanical scalability, graphene has been adopted in the interconnect barrier layer in BEOL at advanced technology nodes, which leads to reduced resistance, suppression of ion migration and enhanced thermal conductivity [310]. For another, graphene based sub-THz receiver holds the potential to be used for the incoming 6G communication, outperforming state-of-the-art sub-THz receiver and mitigating the data traffic issue predicated by Edholms’ law [282]. Due to the intrinsic high surface-to-volume ratio of 2D materials, 2D materials-based sensors are more sensitive and selective. The practical developing road for 2D materials-based electronics is to combine with Si logic circuits to increase the functionality of the chip by concurrently leveraging the merits of modern mature 3D semiconductor technology and the diverse functions of 2D materials-based electronics to obtain more functions to fulfill the increasingly various demands of modern life at the moment. For another, 2D materials-based memristor arrays for computing-in-memory to conquer memory wall or for neuromorphic computing in data-intensive computing demonstrates promising results.

The 2D materials-based ICs are in their nascent stage, enormous challenges and large room for improvements have been still left behind waiting for further endeavors to be devoted to. Fortunately, improvement have been made persistently in all aspects of 2D materials-based ICs, from large-scale synthesis of 2D materials with industry standards and the most “right” 2D materials pursuit, device performance optimization via contact engineering, precisely doping control, high κ dielectric integration, strain engineering to increase carrier mobility, to circuit design optimization to obtain the most functional IC with the least electronic devices to decrease power consumption and increase area-efficiency. Accumulating step and step, one can culminate the mountaintop no matter how high it is. With more and more attention being paid to the development of integrated circuits based on 2D materials, achievements amass to some extent where transformative explosion will break out and 2D materials are poised to give full play to their strength in semiconductor industry some day in the future, either completely take over Si as the dominant semiconductor materials or complement Si to realize their full potential to obtain diverse functions to fulfill the various requirements of modern life.
